# Roles of Altered Macrophages and Cytokines: Implications for Pathological Mechanisms of Postmenopausal Osteoporosis, Rheumatoid Arthritis, and Alzheimer’s Disease

**DOI:** 10.3389/fendo.2022.876269

**Published:** 2022-06-10

**Authors:** Yunteng Xu, Hui Yan, Xin Zhang, Junkuan Zhuo, Yidan Han, Haifeng Zhang, Dingbang Xie, Xin Lan, Wanping Cai, Xiaoning Wang, Shanshan Wang, Xihai Li

**Affiliations:** ^1^ College of Integrative Medicine, Fujian University of Traditional Chinese Medicine, Fuzhou, China; ^2^ Academy of Integrative Medicine, Fujian University of Traditional Chinese Medicine, Fuzhou, China; ^3^ Basic Discipline Laboratory of Integrative Medicine, Fujian University of Traditional Chinese Medicine, Fuzhou, China; ^4^ Key Laboratory of Fujian University of Traditional Chinese Medicine, Fuzhou, China

**Keywords:** postmenopausal osteoporosis, macrophages, cytokines, rheumatoid arthritis, Alzheimer’s disease

## Abstract

Postmenopausal osteoporosis (PMOP) is characterized by the uncoupling of bone resorption and bone formation induced by estrogen deficiency, which is a complex outcome related to estrogen and the immune system. The interaction between bone and immune cells is regarded as the context of PMOP. Macrophages act differently on bone cells, depending on their polarization profile and secreted paracrine factors, which may have implications for the development of PMOP. PMOP, rheumatoid arthritis (RA), and Alzheimer’s disease (AD) might have pathophysiological links, and the similarity of their pathological mechanisms is partially visible in altered macrophages and cytokines in the immune system. This review focuses on exploring the pathological mechanisms of PMOP, RA, and AD through the roles of altered macrophages and cytokines secretion. First, the multiple effects on cytokines secretion by bone-bone marrow (BM) macrophages in the pathological mechanism of PMOP are reviewed. Then, based on the thought of “different tissue-same cell type-common pathological molecules-disease pathological links-drug targets” and the methodologies of “molecular network” in bioinformatics, highlight that multiple cytokines overlap in the pathological molecules associated with PMOP vs. RA and PMOP vs. AD, and propose that these overlaps may lead to a pathological synergy in PMOP, RA, and AD. It provides a novel strategy for understanding the pathogenesis of PMOP and potential drug targets for the treatment of PMOP.

**Graphical Abstract:**
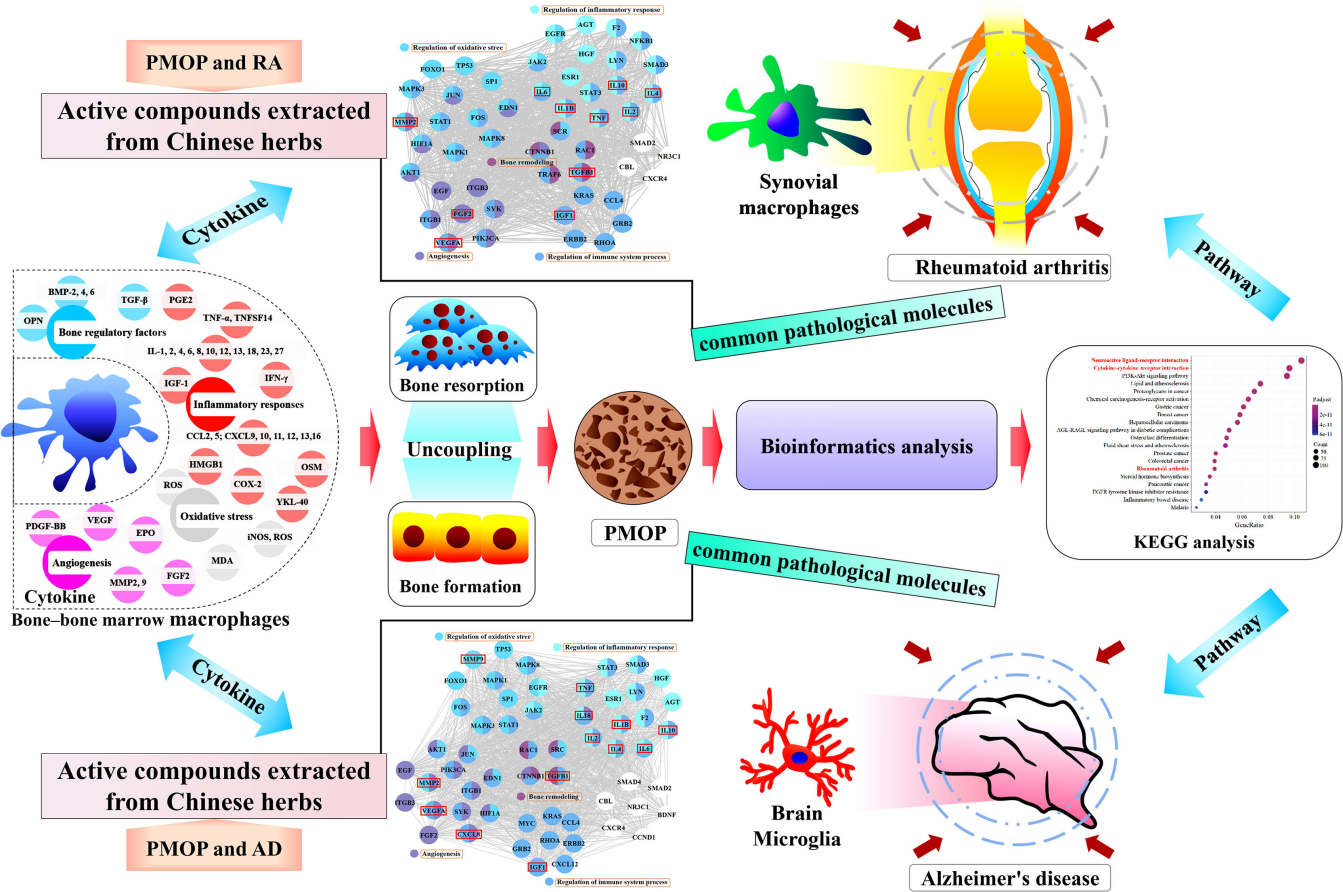


## Introduction

Postmenopausal osteoporosis (PMOP) is a systemic chronic bone metabolic disease caused by the uncoupling of bone resorption and bone formation with estrogen deficiency (1, 2). Estrogen is also involved in the control of immune function, leading to a chronic low-grade pro-inflammatory phenotype under estrogen deficiency with altered cytokine expression and immune cell profiles in PMOP (3–5). Bone and immune system are functionally linked by complex molecular networks, in which accumulating evidence suggests that macrophages either directly or indirectly through the secretion of various cytokines, coordinate the coupling between osteoblasts and osteoclasts (6, 7). The relationship among estrogen, macrophages, and the skeleton could help in understanding the complex mechanism of PMOP.

The complex crosstalk between bone and immune cells plays an indispensable role in the pathogenesis of PMOP. Immunomodulatory imbalances and functional alterations are also part of the pathological conditions of PMOP, rheumatoid arthritis (RA), and Alzheimer’s disease (AD). Immunological studies have demonstrated that different tissue-resident cells of the macrophage lineage, such as bone-bone marrow (BM) macrophages, synovial macrophages, and microglia, are responsible for pathological changes in PMOP, RA, and AD, respectively. The existence of pathological links in PMOP, RA, and AD may be explained by searching for the common molecular network mediated by bone-BM macrophages, synovial macrophages, and microglia to provide a novel strategy for potential drug targets for the treatment of PMOP.

## Diversity of Phenotypes and Functions of Macrophages

Macrophages, which are immune cells with heterogeneous phenotypes and complex functions, can be divided into circulating and resident macrophages (8, 9). Primitive hematopoiesis is a source of macrophages in embryos, and the majority of resident macrophages originate from yolk sac erythro-myeloid progenitors (10, 11). Bone-BM macrophages, synovial macrophages, and microglia play a key role in maintaining tissue homeostasis; phagocytosis and removal of cellular debris and foreign substances; tissue repair, regeneration, and remodeling; and the development and resolution of inflammation (12–15). Under physiological conditions, the bone-BM contains multiple different resident macrophage populations, including osteal macrophages, hematopoietic stem cell niche macrophages, and erythroblast island macrophages (16, 17). Macrophages have remarkable plasticity that allows them to respond efficiently to environmental signals and change their phenotypes.

Macrophages are activated, polarized, and subsequently secreted various cytokines (**Supplementary Table S1**) that are involved in coupling of bone resorption and bone formation under exposure to various types of stimuli. Under exposure to lipopolysaccharide (LPS) or T-helper 1 cytokines, such as interferon-gamma or granulocyte macrophage-colony stimulating factor, alone or in combination, macrophages are activated towards an M1 functional program to produce toxic effector molecules (such as inflammatory cytokines, reactive oxygen, and nitrogen species), which participate in polarized T-helper 1 responses, regulate oxidative stress, and evoke inflammatory responses (18–21). Conversely, T-helper 2 cytokines, such as interleukin (IL)-4 or IL-13, can induce macrophages to polarize into the M2 type including M2a, M2b, M2c, and M2d, and play a central role in polarized T-helper 2 responses, the dampening of inflammation, angiogenesis, immunoregulation, and the remodeling of tissues (11, 22–25). The diversity of the phenotypes and functions of macrophages makes them play important different roles in inflammatory, immune, and metabolic diseases, such as PMOP, RA, and AD.

## Role of Cytokines Secreted by Bone-BM Macrophages in Coupling of Bone Resorption and Bone Formation

### Macrophages Directly Regulate Coupling of Bone Resorption and Bone Formation

Macrophages play a pivotal role in the coupling of bone resorption and bone formation. Paracrine cytokines, such as transforming growth factor-β (TGF-β), bone morphogenetic protein (BMP)-2, BMP-4, BMP-6, and osteopontin, are secreted by activated macrophages, which have a direct and critical impact on the physiological and pathological regulation of bone (**Supplementary Table S2**). On the one hand, fusion between cells of the monocyte/macrophage lineage leads to the formation of osteoclasts, which are the only cells with the ability to dissolve bone tissue (26). On the other hand, ablation of macrophages leads to loss of endosteal osteoblasts, reduction in the number of bone marrow mesenchymal stem cells (BMSCs), decrease in the ability of BMSCs to differentiate into osteoblasts, and attenuation of parathyroid hormone-induced trabecular bone anabolism (27–30). The macrophage-osteoclast axis plays an essential role in osteoimmunity, regulating the coupling of bone resorption and bone formation (31).

### Uncoupling of Bone Resorption and Bone Formation: Cytokines Mediate Inflammatory Responses

Although macrophages in bone-BM are not directly adjacent to osteoblasts, they can alter the BM microenvironment by mediating an inflammatory response to affect bone synthesis and catabolism. Osteocytes are regulated by macrophages that secrete inflammatory mediators to control the coupling of bone resorption and bone formation (**Supplementary Table S3**). Macrophages are vital modulators of inflammation that rapidly change their phenotypes and functions in response to local microenvironmental signals and also play various roles in both the induction and resolution of inflammation, such as clearing dead cells and debris, presenting antigens, and recruiting, and activating other immune cells (7, 32–34). Skeletal homeostasis depends on the balance between the classically active M1 type and the alternatively active M2 type (35, 36). M2 activation appears to be blunted in macrophages from postmenopausal women, leading to an increased M1/M2 response ratio (32). In ovariectomized (OVX) mice, polarization of M1 macrophages was increased whereas polarization of M2 macrophages was disturbed (37). Therefore, changes in macrophage-derived cytokines and their phenotypes linked with inflammation are critical regulators of bone resorption and bone formation, supporting the theory that the immune system significantly contributes to the pathological mechanism of inflammation-mediated bone-loss.

### Uncoupling of Bone Resorption and Bone Formation: Cytokines Mediate Oxidative Stress

After menopause, due to the influence of estrogen deficiency, the level of oxidative stress in the body increases, which causes the imbalance in bone reconstruction and leads to osteoporosis (38–40). Macrophages secrete regulatory factors related to oxidative stress, such as reactive oxygen species (ROS), nitric oxide (NO), and inducible nitric oxide synthase, which induce pathological changes in the differentiation process and activity of bone cells, ultimately leading to the uncoupling of bone resorption and bone formation (**Supplementary Table S4**). The oxidative stress level in PMOP depends on the relationship between ROS and the endogenous antioxidant defense system (41, 42). One of the most damaging effects of ROS is lipid peroxidation, whose end product, malondialdehyde, is a potential biomarker of oxidative stress (43). NO, catalyzed by nitric oxide synthase, is also an integral part of the response to oxygen deprivation and has been confirmed to be a key regulator of bone homeostasis (44–46). The effects of these factors highlight the diversity of the roles of macrophages in regulating bone homeostasis. Further studies are needed to clarify the molecular mechanisms underlying the relationship among macrophages, oxidative stress and PMOP.

### Uncoupling of Bone Resorption and Bone Formation: Cytokines Mediate Angiogenesis

Bone is a highly vascularized tissue, and bone homeostasis depends on the coupling between bone and blood vessels. The skeletal microvasculature system plays an important role in the metabolism of BM microenvironment, osteogenesis, and maintenance of the balance between bone formation and bone resorption. Basic and clinical studies have found that the decrease in local blood supply is related to PMOP. In the OVX mouse model, the number of microvessels, the type H vessels, and the expression of vascular endothelial growth factor (VEGF) are all significantly reduced (47, 48). Macrophages are key cellular components in the BM microenvironment that regulate bone homeostasis and angiogenesis. In bone repair, macrophages can remove dead neutrophils at the injured site after fracture, and release cytokines, such as VEGF, erythropoietin, platelet-derived growth factor-BB, matrix metallopeptidase 2 (MMP2), MMP9, and fibroblast growth factor 2 (FGF2) (**Supplementary Table S5**), so as to initiate the repair cascade that suppresses the pro-inflammatory responses and promotes angiogenic responses (49, 50). During the inflammatory phase of bone repair, the recruitment of macrophages is related to angiogenesis, and their numbers are strongly correlated with the density of blood vessels (51). Moreover, the coordinated conversion of the pro-inflammatory M1 and anti-inflammatory M2 phenotypes in macrophages determines the efficiency of bone regeneration to a great extent (52). Given their intimate involvement in vascular formation, an understanding of the multilayered contributions of macrophages to bone repair and fracture healing is also accumulating.

## Bioinformatics Identified Shared Pathological Molecules in PMOP, RA, and AD

### Bioinformatics Revealed Potential Pathological Links in PMOP, RA, and AD

**Figure 1:**
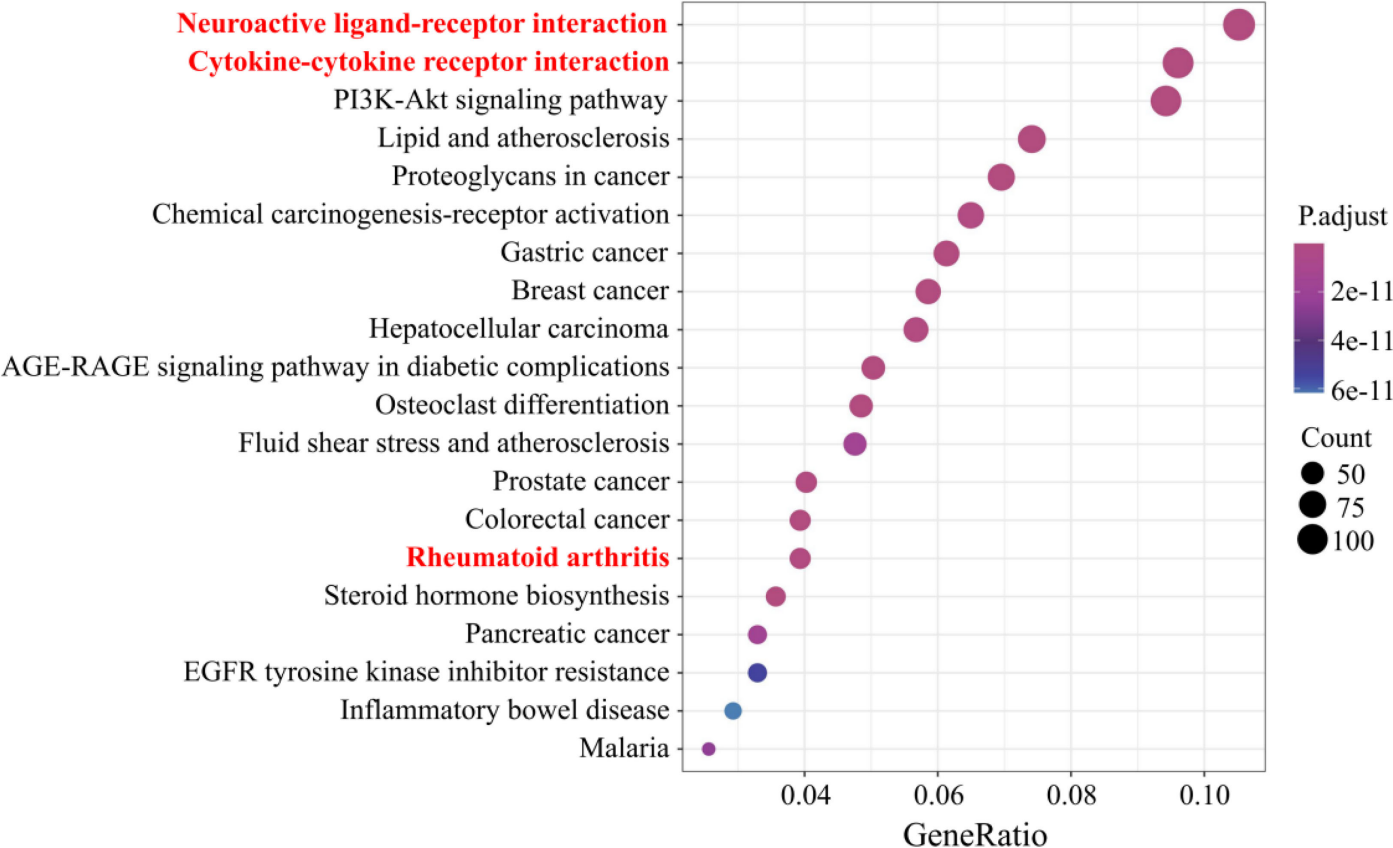
Top 20 Kyoto Encyclopedia of Genes and Genomes (KEGG) pathway enrichment candidate targets of differential genes in postmenopausal osteoporosis. Pathways with significant changes (false discovery rate [FDR] < 0.05) were identified. The vertical coordinates represent the KEGG pathway with significant enrichment, and the horizontal coordinates represent the gene ratio, which refers to the ratio of enriched genes to all target genes. The color of the bubble graph indicates the significance of the enriched KEGG pathway, the color gradient represents the size of the *P*-value, and the size of each dot represents the number of genes.

Because the physiological and immune functions are reduced in postmenopausal women, in addition to the need to prevent osteoporosis, the prevalence of RA and AD is also quite noteworthy. A large number of clinical and basic studies have confirmed the association in PMOP, RA, and AD. With the help of bioinformatics analysis methods (**Supplementary Methods of Bioinformatics**), we integrated multiple databases to screen the differential genes of PMOP, and then performed enrichment analysis of the Kyoto Encyclopedia of Genes and Genomes (KEGG) pathway, which was also enriched in the RA and AD pathways (**Figure 1** and **Table 1**). Simultaneously, the two signaling pathways of neuroactive ligand-receptor interaction and cytokine-cytokine receptor interaction also undergo significant changes. Like PMOP, the pathological mechanisms of RA and AD are also closely associated with two resident macrophages: synovial macrophages and brain microglia, respectively. Therefore, we pose the question of what role do macrophages play in the “two-pairs of disease links”. Searching for significant common-targets in PMOP, RA, and AD may have a particularly practical meaning in providing guidance for the prevention and control of PMOP, RA, and AD.

**Table 1:** Results of KEGG enrichment analysis of RA and AD pathways.

Description	Gene Ratio	Bg Ratio	*P-*value	*P*-adjust	*Q-*value
Rheumatoid arthritis (hsa05323)	43/1093	93/8112	1.25E-14	3.13E-13	1.48E-13
Alzheimer’s disease (hsa05010)	66/1093	384/8112	0.019899217	0.039433384	0.01871717

### Common Pathological Molecules Between PMOP and RA: A Molecular Perspective of Cytokines Secreted From Bone-BM Macrophages and Synovial Macrophages

The immune cells involved in RA, macrophages, are the most numerous immune cells found in the RA synovium and play a key role in immune/inflammatory reactions and bone loss by paracrine signaling or *via* direct cell-cell contact (53–56). In addition, synovial macrophages are involved in pathological processes such as matrix degradation, oxidative stress, and angiogenesis in RA (57–59).

Among other features, RA is characterized by systemic bone loss, and the risk of osteoporosis is high in patients with RA, especially in postmenopausal women (60–62). Therefore, to clarify the pathological links between PMOP and RA, we used bioinformatics analysis methods (**Supplementary Methods of Bioinformatics**) to search for common pathological molecules between them.

**Figure 2:**
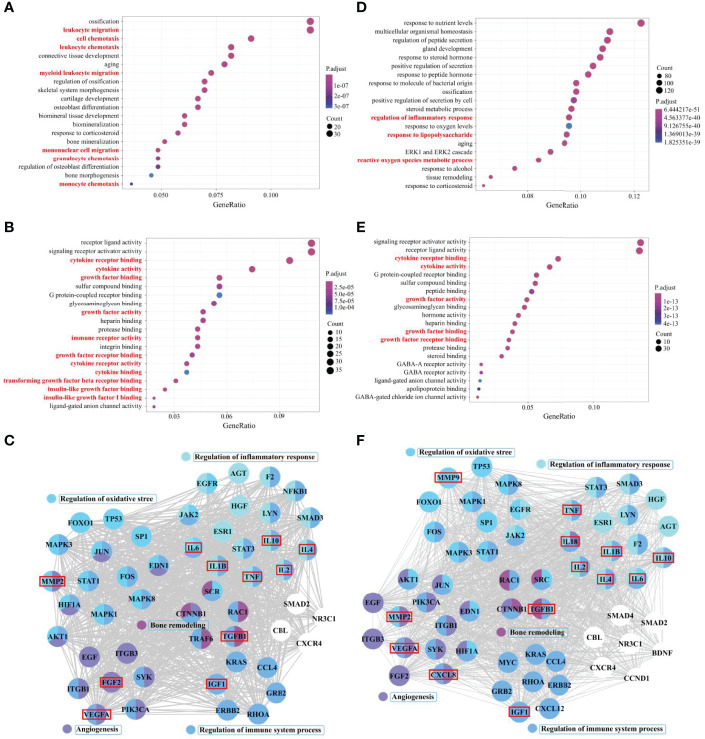
Core cytokine networks of pathological crosstalk in postmenopausal osteoporosis (PMOP) vs. rheumatoid arthritis (RA) and PMOP vs. Alzheimer’s disease (AD). Gene ontology functional enrichment analysis of common differential genes in PMOP vs. RA and PMOP vs. AD was performed, including biological processes **(A, D)** and molecular functions **(B, E)**. Protein–protein interaction (PPI) network topology analysis was performed for common differential genes in PMOP vs. RA and PMOP vs. AD, and biological process enrichment analysis of core network genes was completed **(C, F)**.

The results are summarized as follows: among biological processes, it is enriched in leukocyte migration, cell chemotaxis, leukocyte chemotaxis, myeloid, mononuclear cell migration, granulocyte chemotaxis, monocyte chemotaxis, and other processes. (**Figure 2A**). Among molecular functions, it is enriched in cytokine receptor binding, cytokine activity, growth factor binding, growth factor activity, immune receptor activity, growth factor receptor binding, cytokine receptor activity, cytokine binding, TGF-β receptor binding, insulin-like growth factor (IGF) binding, IGF-1 binding, and other functions (**Figure 2B**). The results show that the immune system and immune cells play important regulatory roles in the occurrence of PMOP and RA. Although the results did not directly enrich macrophage-related functions in the top 20 Gene Ontology functions, the related functions enriched in cells (such as TGF-β and IGF-1) still had great directivity. Cytokines secreted by macrophages, including TGF-β1, IL-1β, IL-2, IL-4, IL-6, IL-10, tumor necrosis factor (TNF), IGF-1, VEGFA, FGF2 and MMP2, which are associated with the functions of regulation of immune system process, bone remodeling, regulation of inflammatory response, response to oxidative stress, and angiogenesis, were screened from the protein–protein interaction (PPI) core network, as described above (**Figure 2C**).

As in PMOP, an imbalanced network of cytokines secreted by synovial macrophages plays a key role in the pathogenesis of RA. Among them, secretion of TNF-α, IL-1β, IL-2, and IL-6, or a combined deficiency of IL-4 and IL-10, promotes and sustains inflammation, while also acting to promote bone erosion (63, 64). In addition, TGF-β, TNF-α, IGF-1, VEGF, FGF2, and MMP2 are involved in the hypervascularization as well as the pannus formation observed in RA (65–67). As a result, the imbalanced cytokine network could provide clues to identify pathological links between the two diseases and potentially suggest some shared pharmacological prevention and treatment.

### Common Pathological Molecules between PMOP and AD: A Molecular Perspective of Cytokines Secreted From Bone-BM Macrophages and Microglia

Microglia may play a significant role in the pathogenesis of AD, which is characterized by deposition of β-amyloid plaques, hyperphosphorylation of tau protein, oxidative damage, neuroinflammation, vascular remodeling, autophagy, and mitochondrial dysfunction (68–71). AD and PMOP are frequently seen to coincide in clinical practice, and their possible relationship, concurrent occurrence, and linking mechanism have recently been highlighted (72–74). Prevention of osteoporosis should be considered as part of the treatment of patients with AD, especially in postmenopausal women, and conversely, prevention of AD should be considered in patients with various degrees of bone loss.

Microglia show altered morphology and reduced arborization, and their activation increases with the progression of AD (75, 76). Activated microglia exhibit many morphologic and immunophenotypic features of peripheral macrophages, such as pro-inflammatory M1 and immunosuppressive M2 phenotypes (77, 78). Activated microglia assume diverse phenotypes, which mediate the different pathological processes of AD by releasing various substances, such as inflammatory cytokines, growth factors, chemokines, neurotrophins, and superoxide (79–82).

We also used bioinformatics analysis methods (**Supplementary Methods of Bioinformatics**) to search for common pathological molecules between PMOP and AD. Among biological processes, it’s enriched in the regulation of inflammatory response, response to LPS, ROS metabolic process, and other processes (**Figure 2D**). Among molecular function, it is enriched in the cytokine receptor binding, cytokine activity, growth factor activity, growth factor binding and receptor binding, and other functions (**Figure 2E**). Cytokines secreted by macrophages, including TGF-β1, IL-1β, IL-2, IL-4, IL-6, IL-10, IL-18, TNF, IGF-1, C-X-C motif chemokine ligand 8 (CXCL8), VEGFA, FGF2, MMP2, and MMP9, which are also associated with the functions of regulation of immune system process, bone remodeling, regulation of inflammatory response, response to oxidative stress, and angiogenesis, were screened from the PPI core network, as described above (**Figure 2F**). The results indicated that the pathological changes in bone-BM macrophage-mediated PMOP are partially similar to the pathological changes in microglia-mediated AD.

The delicate balance between their pro-inflammatory and anti-inflammatory actions and their neurotoxic and neuroprotective actions determines the role of microglia in AD. Microglia activate and drive inflammatory processes by inducing the pro-inflammatory molecules, such as IL-1β, IL-6, IL-18, and TNF-α, leading to accumulation of extracellular amyloid-β peptides, tau hyperphosphorylation, and activation of other inflammatory participants (83, 84). They also produce various anti-inflammatory, chemokines and growth factors, such as IL-2, IL-4, IL-10, CXCL8, FGF2, IGF-1, and TGF-β1, which have been shown to exert neuroprotective effects against amyloid-β-induced neurodegeneration (85–88). Other microglia-derived factors such as VEGFA, MMP2, and MMP9, are associated with disruption of the blood-brain barrier, leading to neuroinflammation and progression of AD (89–91). Combined with the role of macrophages in PMOP mentioned above, simultaneous tracing of the common pathological molecular network associated with cytokines (bone-BM macrophages and microglia) in PMOP and AD may reveal the key pathological links between the two diseases.

## Exploration of Multifunctional Potential Active Components From Chinese Herbs Targeting Common Pathological Molecules of PMOP, RA, and AD

Because of the common pathological molecules of PMOP, RA, and AD, it is of great importance to seek effective drugs to prevent the occurrence of complications. Chinese herbs have anti-PMOP, anti-RA and anti-AD properties due to their actions against multiple targets, pathways, and systems. Therefore, taking the cytokines secreted by macrophages as the entry point, combined with the results of bioinformatics analysis, we summarized potential active components extracted from Chinese herbs, such as icariin, querzcetin, and naringin, which were simultaneously applied in the treatment of PMOP, RA, and AD (**Tables 2** and **3**). From the side, this also reflects the roles of altered macrophages and cytokines on PMOP, RA, and AD.

**Table 2:** Summary of potential active components from Chinese herbs to be applied in PMOP and RA.

Active component from Chinese herbs	Targets	Pharmacodynamic mechanism in PMOP	Ref	Pharmacodynamic mechanism in RA	Ref
Icariin	IL-6	a. Diminished LPS induced IL-6 and TNF-α on osteoclasts, and decreased PGE2 production by inhibiting COX-2. b. Inhibited IL-1β in OVX rats. c. Reduced MMP-9 in RANKL-induced osteoclast formation from RAW 264.7 cells. d. Reduced MDA in hypoxia-induced oxidative damage of osteoblasts.	(92–94)	a. Inhibited IL-6, TNF-a, and IL-1β in RA-FLS cells. b. Wangbi capsule, whose main effective substances include icariin, reduced PGE2 and IL-1β in adjuvant induced arthritis rat model. c. Inhibited MMP in induction of type II collagen-induced arthritis. d. Reduced MDA levels in LPS-induced synovitis.	(95–98)
	IL-1β				
	TNF-α				
	PGE2				
	MMP9				
Luteolin	NO	a. Decreased the 3-morpholinosydnonimie-induced production of NO, TNF-a, and IL-6 in osteoblasts.	(99)	a. Reduced NO, TNF-α, and IL-6 in LPS-induced RAW 264.7 macrophages and ConA-induced T lymphocytes.	(100)
	TNF-α				
	IL-6				
Quercetin	TNF-α	a. Significantly decreased TNF-α in OVX rat model. b. Reduced IL-6 and TNF-α in RANKL-induced osteoclasts.	(101, 102)	a. Down-regulated the content of TNF-α, IL-1β and IL-6 in collagen-induced arthritis mice.	(103)
	IL-6				
Naringin	NO	a. Enhanced NO synthesis in OVX rat model. b. Prevented TNF-α-inhibited BMSCs osteogenic differentiation of BMSCs.	(104, 105)	a. All flavonoids, including naringin, inhibited NO production from LPS-induced macrophage cells. b. Inhibited IL-6 and IL-1β in TNF-α-induced RA-FLS.	(106, 107)
	TNF-α				

**Table 3:** Summary of potential active components from Chinese herbs to be applied in PMOP and RA.

Active component from Chinese herbs	Targets	Pharmacodynamic mechanism in PMOP	Ref	Pharmacodynamic mechanism in AD	Ref
Icariin	COX-2	a. Inhibited LPS-induced bone resorption and TNF-α expression, also inhibited COX-2 and PGE2 synthesis on osteoblasts or osteoclasts. b. Increased NO production in BMSCs and osteoblasts, and inhibited osteoclast-mediated bone resorption. c. Reduced production of ROS and MDA in osteoblasts.	(92, 94, 108, 109)	a. Decreased expression of TNF-α and COX-2 in hippocampus of rats with LPS-induced brain dysfunction. b. Inhibited the release of ROS, NO, and PGE2 in microglia. c. Reduced MDA content in hippocampus of aluminum-poisoned rats.	(110–112)
	PEG2				
	TNF-α				
	NO				
	MDA				
	ROS				
Naringin	NO	a. Enhanced NO synthesis in OVX rats. b .TNF-α: as shown in **Table 2**.	(104, 105)	a. Reduced hippocampal NO production in a mouse model of AD. b. Reduced TNF-α levels in ICV-STZ rats.	(113, 114)
	TNF-α				
Quercetin	ROS TNF-α	a. Protected against TNF-α-induced impairments in BMSCs osteogenesis. b. Reduced ROS and TNF-α levels when coculturing osteoblast-osteoclast or triculturing osteoblast-osteoclast-endothelial cells on hydroxyapatite loaded with quercetin.	(115, 116)	a. Reduced ROS and TNF-α levels in high-cholesterol-fed aged mice. b. Reduced TNF-α and -IL-6 expression and reversed neurodegeneration to restore memory function.	(117, 118)

## Conclusions and Perspectives

PMOP is caused by dysregulation of the homeostatic connection between bone and the immune system, leading to bone loss. This review has outlined the direct and indirect effects of cytokines secreted by bone-BM macrophages on the coupling of bone resorption and bone formation. The principal mechanisms of these effects include inflammatory/immune responses, angiogenesis, and oxidative stress. Some overlapping cytokines of PMOP, RA, and AD in bioinformatics analysis may immunologically link two diseases, serving as either shared susceptibility factors or molecular links. Therefore, based on the thought of “different tissue (bone-BM, synovial, and brain)-same cell type (macrophages)-common pathological molecules (cytokines)-disease pathological links (PMOP vs. RA and PMOP vs. AD)-drug targets (active compounds extracted from Chinese herbs)” and the methodologies of “molecular network” in bioinformatics, may lead to a paradigm shift in the understanding of the pathogenesis, prophylaxis, and treatment of PMOP.

## Author Contributions

XHL and YX identified the focus and overall direction of the review. Funding acquisition XHL and HY; Sources, XHL; Methodology, XZ, JZ, YH, HZ, DX, XL, WC, XW, SW, and XHL; Supervision, XHL; Writing—original draft, YX, HY, and XHL; Writing—review & editing, YX, HY, XZ, JZ, YH, HZ, DX, XL, WC, XW, SW, and XHL. All authors contributed to the article and approved the submitted version.

## Funding

This work was funded by the National Natural Science Foundation of China (No. 82074461), the Chen Keji Development Fund of Integrative Medicine (No. 2020004) and the Research Start-up Fund of Fujian University of Traditional Chinese Medicine (No. X2020009-Talent).

## Conflict of Interest

The authors declare that the research was conducted in the absence of any commercial or financial relationships that could be construed as a potential conflict of interest.

## Publisher’s Note

All claims expressed in this article are solely those of the authors and do not necessarily represent those of their affiliated organizations, or those of the publisher, the editors and the reviewers. Any product that may be evaluated in this article, or claim that may be made by its manufacturer, is not guaranteed or endorsed by the publisher.

## References

[1] Møller A Delaissé JM Olesen JB Madsen JS Canto LM Bechmann T. Aging and Menopause Reprogram Osteoclast Precursors for Aggressive Bone Resorption. Bone Res (2020) 8:27–37. doi: 10.1038/s41413-020-0102-7 32637185 PMC7329827

[2] Pacifici R. Estrogen, Cytokines, and Pathogenesis of Postmenopausal Osteoporosis. J Bone Miner Res (1996) 11(8):1043–51. doi: 10.1002/jbmr.5650110802 8854239

[3] Khosla S Oursler MJ Monroe DG. “Estrogen and the Skeleton”. Trends Endocrinol Metab (2012) 23(11):576–81. doi: 10.1016/j.tem.2012.03.008 PMC3424385 22595550

[4] Ralston SH. Analysis of Gene Expression in Human Bone Biopsies by Polymerase Chain Reaction: Evidence for Enhanced Cytokine Expression in Postmenopausal Osteoporosis. J Bone Miner Res (1994) 9(6):883–90. doi: 10.1002/jbmr.5650090614 8079663

[5] D’Amelio P. The Immune System and Postmenopausal Osteoporosis. Immunol Invest (2013) 42(7):544–54. doi: 10.3109/08820139.2013.822764 24004058

[6] Yang DH Yang MY. The Role of Macrophage in the Pathogenesis of Osteoporosis. Int J Mol Sci (2019) 20(9):2093–107. doi: 10.3390/ijms20092093 PMC6539137 31035384

[7] Muñoz J Akhavan NS Mullins AP Arjmandi BH. Macrophage Polarization and Osteoporosis: A Review. Nutrients (2020) 12(10):2999–3104. doi: 10.3390/nu12102999 PMC7601854 33007863

[8] Guilliams M Scott CL. Does Niche Competition Determine the Origin of Tissue-Resident Macrophages? Nat Rev Immunol (2017) 17(7):451–60. doi: 10.1038/nri.2017.42 28461703

[9] Okabe Y Medzhitov R. Tissue Biology Perspective on Macrophages. Nat Immunol (2016) 17(1):9–17. doi: 10.1038/ni.3320 26681457

[10] Perdiguero EG Geissmann F. The Development and Maintenance of Resident Macrophages. Nat Immunol (2016) 17(1):2–8. doi: 10.1038/ni.3341 26681456 PMC4950995

[11] Franken L Schiwon M Kurts C. Macrophages: Sentinels and Regulators of the Immune System. Cell Microbiol (2016) 18(4):475–87. doi: 10.1111/cmi.12580 26880038

[12] Casanova-Acebes M Dalla E Leader AM LeBerichel J Nikolic J Morales BM. Tissue-Resident Macrophages Provide a Pro-Tumorigenic Niche to Early NSCLC Cells. Nature (2021) 595(7868):578–84. doi: 10.1038/s41586-021-03651-8 PMC8923521 34135508

[13] Italiani P Boraschi D. New Insights Into Tissue Macrophages: From Their Origin to the Development of Memory. Immune Netw (2015) 15(4):167–76. doi: 10.4110/in.2015.15.4.167 PMC4553254 26330802

[14] Wynn TA Vannella KM. Macrophages in Tissue Repair, Regeneration, and Fibrosis. Immunity (2016) 44(3):450–62. doi: 10.1016/j.immuni.2016.02.015 PMC4794754 26982353

[15] Dey A Allen J Hankey-Giblin PA. Ontogeny and Polarization of Macrophages in Inflammation: Blood Monocytes Versus Tissue Macrophages. Front Immunol (2014) 5:683. doi: 10.3389/fimmu.2014.00683 25657646 PMC4303141

[16] Chen K Jiao Y Liu L Huang M He C He W. Communications Between Bone Marrow Macrophages and Bone Cells in Bone Remodeling. Front Cell Dev Biol (2020) 8:598263. doi: 10.3389/fcell.2020.598263 33415105 PMC7783313

[17] Kaur S Raggatt LJ Batoon L Hume DA Levesque JP Pettit AR. Role of Bone Marrow Macrophages in Controlling Homeostasis and Repair in Bone and Bone Marrow Niches. Semin Cell Dev Biol (2017) 61:12–21. doi: 10.1016/j.semcdb.2016.08.009 27521519

[18] Martinez FO Gordon S. The M1 and M2 Paradigm of Macrophage Activation: Time for Reassessment. F1000Prime Rep (2014) 6:13–25. doi: 10.12703/P6-13 24669294 PMC3944738

[19] Muraille E Leo O Moser M. TH1/TH2 Paradigm Extended: Macrophage Polarization as an Unappreciated Pathogen-Driven Escape Mechanism? Front Immunol (2014) 5:603. doi: 10.3389/fimmu.2014.00603 25505468 PMC4244692

[20] Fresta CG Fidilio A Lazzarino G Musso N Grasso M Merlo S. Modulation of Pro-Oxidant and Pro-Inflammatory Activities of M1 Macrophages by the Natural Dipeptide Carnosine. Int J Mol Sci (2020) 21(3):776–97. doi: 10.3390/ijms21030776 PMC7038063 31991717

[21] Karkossa I Raps S von Bergen M Schubert K. Systematic Review of Multi-Omics Approaches to Investigate Toxicological Effects in Macrophages. Int J Mol Sci (2020) 21(24). doi: 10.3390/ijms21249371 PMC7764599 33317022

[22] Shapouri-Moghaddam A Mohammadian S Vazini H Taghadosi M Esmaeili SA Mardani F. Macrophage Plasticity, Polarization, and Function in Health and Disease. J Cell Physiol (2018) 233(9):6425–40. doi: 10.1002/jcp.26429 PMC13482560 29319160

[23] Sica A Mantovani A. Macrophage Plasticity and Polarization: *In Vivo* Veritas. J Clin Invest (2012) 122(3):787–95. doi: 10.1172/JCI59643 PMC3287223 22378047

[24] Jetten N Verbruggen S Gijbels MJ Post MJ De Winther MP Donners MM. Anti-Inflammatory M2, But Not Pro-Inflammatory M1 Macrophages Promote Angiogenesis *In Vivo* . Angiogenesis (2014) 17(1):109–18. doi: 10.1007/s10456-013-9381-6 24013945

[25] Ramirez-GarciaLuna JL Rangel-Berridi K Olasubulumi OO Rosenzweig DH Henderson JE Gawri R. Enhanced Bone Remodeling After Fracture Priming. Calcif Tissue Int (2021) 110(3):349–66. doi: 10.1007/s00223-021-00921-5 34668029

[26] Pereira M Petretto E Gordon S Bassett J Williams GR Behmoaras J. Common Signalling Pathways in Macrophage and Osteoclast Multinucleation. J Cell Sci (2018) 131(11):jcs216267. doi: 10.1242/jcs.216267 29871956

[27] Winkler IG Sims NA Pettit AR Barbier V Nowlan B Helwani F. Bone Marrow Macrophages Maintain Hematopoietic Stem Cell (HSC) Niches and Their Depletion Mobilizes HSCs. Blood (2010) 116(23):4815–28. doi: 10.1182/blood-2009-11-253534 20713966

[28] Jacobsen RN Forristal CE Raggatt LJ Nowlan B Barbier V Kaur S. Mobilization With Granulocyte Colony-Stimulating Factor Blocks Medullar Erythropoiesis by Depleting F4/80(+)VCAM1(+)CD169(+)ER-HR3(+)Ly6G(+) Erythroid Island Macrophages in the Mouse. Exp Hematol (2014) 42(7):547–61.e4. doi: 10.1016/j.exphem.2014.03.009 24721610

[29] Vi L Baht GS Whetstone H Ng A Wei Q Poon R. Macrophages Promote Osteoblastic Differentiation *in-Vivo*: Implications in Fracture Repair and Bone Homeostasis. J Bone Miner Res (2015) 30(6):1090–102. doi: 10.1002/jbmr.2422 25487241

[30] Cho SW Soki FN Koh AJ Eber MR Entezami P Park SI. Osteal Macrophages Support Physiologic Skeletal Remodeling and Anabolic Actions of Parathyroid Hormone in Bone. Proc Natl Acad Sci USA (2014) 111(4):1545–50. doi: 10.1073/pnas.1315153111 PMC3910564 24406853

[31] Yao Y Cai X Ren F Ye Y Wang F Zheng C. The Macrophage-Osteoclast Axis in Osteoimmunity and Osteo-Related Diseases. Front Immunol (2021) 12 664871:664871. doi: 10.3389/fimmu.2021.664871 33868316 PMC8044404

[32] Fischer V Haffner-Luntzer M. Interaction Between Bone and Immune Cells: Implications for Postmenopausal Osteoporosis. Semin Cell Dev Biol (2021)123:14–21. doi: 10.1016/j.semcdb.2021.05.014 34024716

[33] Tysoe O. Osteal Macrophages Implicated in Osteoporosis. Nat Rev Endocrinol (2021) 17(10):577. doi: 10.1038/s41574-021-00548-4 34316044

[34] Schlundt C Fischer H Bucher CH Rendenbach C Duda GN Schmidt-Bleek K. The Multifaceted Roles of Macrophages in Bone Regeneration: A Story of Polarization, Activation and Time. Acta Biomater (2021) 133:46–57. doi: 10.1016/j.actbio.2021.04.052 33974949

[35] Sun Y Li J Xie X Gu F Sui Z Zhang K. Macrophage-Osteoclast Associations: Origin, Polarization, and Subgroups. Front Immunol (2021) 12:778078. doi: 10.3389/fimmu.2021.778078 34925351 PMC8672114

[36] Gong L Zhao Y Zhang Y Ruan Z. The Macrophage Polarization Regulates MSC Osteoblast Differentiation *In Vitro* . Ann Clin Lab Sci (2016) 46(1):65–71. 26927345

[37] Toniolo A Fadini GP Tedesco S Cappellari R Vegeto E Maggi A. Alternative Activation of Human Macrophages Is Rescued by Estrogen Treatment *In Vitro* and Impaired by Menopausal Status. J Clin Endocrinol Metab (2015) 100(1):E50–8. doi: 10.1210/jc.2014-2751 25303489

[38] Manolagas SC. From Estrogen-Centric to Aging and Oxidative Stress: A Revised Perspective of the Pathogenesis of Osteoporosis. Endocr Rev (2010) 31(3):266–300. doi: 10.1210/er.2009-0024 20051526 PMC3365845

[39] Banfi G Iorio EL Corsi MM. Oxidative Stress, Free Radicals and Bone Remodeling. Clin Chem Lab Med (2008) 46(11):1550–5. doi: 10.1515/CCLM.2008.302 18847368

[40] Mohamad NV Ima-Nirwana S Chin KY. Are Oxidative Stress and Inflammation Mediators of Bone Loss Due to Estrogen Deficiency? A Review of Current Evidence. Endocr Metab Immune Disord Drug Targets (2020) 20(9):1478–87. doi: 10.2174/1871530320666200604160614 PMC8383467 32496996

[41] Wauquier F Leotoing L Coxam V Guicheux J Wittrant Y. Oxidative Stress in Bone Remodelling and Disease. Trends Mol Med (2009) 15(10):468–77. doi: 10.1016/j.molmed.2009.08.004 19811952

[42] Kimball JS Johnson JP Carlson DA. Oxidative Stress and Osteoporosis. J Bone Joint Surg Am (2021) 103(15):1451–61. doi: 10.2106/JBJS.20.00989 34014853

[43] Barrera G Pizzimenti S Daga M Dianzani C Arcaro A Cetrangolo GP. Lipid Peroxidation-Derived Aldehydes, 4-Hydroxynonenal and Malondialdehyde in Aging-Related Disorders. Antioxid (Basel) (2018) 7(8). doi: 10.3390/antiox7080102 PMC6115986 30061536

[44] Blokhina O Fagerstedt KV. Oxidative Metabolism, ROS and NO Under Oxygen Deprivation. Plant Physiol Biochem (2010) 48(5):359–73. doi: 10.1016/j.plaphy.2010.01.007 20303775

[45] Joshua J Kalyanaraman H Marathe N Pilz RB. Nitric Oxide as a Mediator of Estrogen Effects in Osteocytes. Vitam Horm (2014) 96:247–63. doi: 10.1016/B978-0-12-800254-4.00010-6 25189390

[46] Wimalawansa SJ. Nitric Oxide and Bone. Ann NY Acad Sci (2010) 1192:391–403. doi: 10.1111/j.1749-6632.2009.05230.x 20392265

[47] Ding WG Wei ZX Liu JB. Reduced Local Blood Supply to the Tibial Metaphysis Is Associated With Ovariectomy-Induced Osteoporosis in Mice. Connect Tissue Res (2011) 52(1):25–9. doi: 10.3109/03008201003783011 20497029

[48] Wang L Ma R Guo Y Sun J Liu H Zhu R. Antioxidant Effect of Fructus Ligustri Lucidi Aqueous Extract in Ovariectomized Rats Is Mediated Through Nox4-ROS-NF-κb Pathway. Front Pharmacol (2017) 8:266. doi: 10.3389/fphar.2017.00266 28588482 PMC5438993

[49] Claes L Recknagel S Ignatius A. Fracture Healing Under Healthy and Inflammatory Conditions. Nat Rev Rheumatol (2012) 8(3):133–43. doi: 10.1038/nrrheum.2012.1 22293759

[50] Hu K Olsen BR. Vascular Endothelial Growth Factor Control Mechanisms in Skeletal Growth and Repair. Dev Dyn (2017) 246(4):227–34. doi: 10.1002/dvdy.24463 PMC5354946 27750398

[51] Hu K Olsen BR. Osteoblast-Derived VEGF Regulates Osteoblast Differentiation and Bone Formation During Bone Repair. J Clin Invest (2016) 126(2):509–26. doi: 10.1172/JCI82585 PMC4731163 26731472

[52] Zheng Z Chen Y Hong H Shen Y Wang Y Sun J. The “Yin and Yang” of Immunomodulatory Magnesium-Enriched Graphene Oxide Nanoscrolls Decorated Biomimetic Scaffolds in Promoting Bone Regeneration. Adv Healthc Mater (2021) 10(2):e2000631. doi: 10.1002/adhm.202000631 33166076

[53] Ardura JA Rackov G Izquierdo E Alonso V Gortazar AR Escribese MM. Targeting Macrophages: Friends or Foes in Disease? Front Pharmacol (2019) 10:1255. doi: 10.3389/fphar.2019.01255 31708781 PMC6819424

[54] Chen Z Bozec A Ramming A Schett G. Anti-Inflammatory and Immune-Regulatory Cytokines in Rheumatoid Arthritis. Nat Rev Rheumatol (2019) 15(1):9–17. doi: 10.1038/s41584-018-0109-2 30341437

[55] Tardito S Martinelli G Soldano S Paolino S Pacini G Patane M. Macrophage M1/M2 Polarization and Rheumatoid Arthritis: A Systematic Review. Autoimmun Rev (2019) 18(11):102397. doi: 10.1016/j.autrev.2019.102397 31520798

[56] Kinne RW Bräuer R Stuhlmüller B Palombo-Kinne E Burmester GR. Macrophages in Rheumatoid Arthritis. Arthritis Res (2000) 2(3):189–202. doi: 10.1186/ar86 11094428 PMC130001

[57] Elshabrawy HA Chen Z Volin MV Ravella S Virupannavar S Shahrara S. The Pathogenic Role of Angiogenesis in Rheumatoid Arthritis. Angiogenesis (2015) 18(4):433–48. doi: 10.1007/s10456-015-9477-2 PMC4879881 26198292

[58] Yang Z Shen Y Oishi H Matteson EL Tian L Goronzy JJ. Restoring Oxidant Signaling Suppresses Proarthritogenic T Cell Effector Functions in Rheumatoid Arthritis. Sci Transl Med (2016) 8(331):331ra38. doi: 10.1126/scitranslmed.aad7151 PMC5074090 27009267

[59] Szekanecz Z Koch AE. Macrophages and Their Products in Rheumatoid Arthritis. Curr Opin Rheumatol (2007) 19(3):289–95. doi: 10.1097/BOR.0b013e32805e87ae 17414958

[60] Sapir-Koren R Livshits G. Postmenopausal Osteoporosis in Rheumatoid Arthritis: The Estrogen Deficiency-Immune Mechanisms Link. Bone (2017) 103:102–15. doi: 10.1016/j.bone.2017.06.020 28666971

[61] Haugeberg G Uhlig T Falch JA Halse JI Kvien TK. Bone Mineral Density and Frequency of Osteoporosis in Female Patients With Rheumatoid Arthritis: Results From 394 Patients in the Oslo County Rheumatoid Arthritis Register. Arthritis Rheum (2000) 43(3):522–30. doi: 10.1002/1529-0131(200003)43:3<522::AID-ANR7>3.0.CO;2-Y 10728744

[62] Mirzaei A Jahed SA Amini KA Zabihiyeganeh M. Risk of Infection in Postmenopausal Women With Rheumatoid Arthritis and Osteoporosis Taking Denosumab and bDMARDS. Med J Islam Repub Iran (2021) 35(12):12–5. doi: 10.47176/mjiri.35.12 PMC8111619 33996663

[63] Boutet MA Courties G Nerviani A Le Goff B Apparailly F Pitzalis C. Novel Insights Into Macrophage Diversity in Rheumatoid Arthritis Synovium. Autoimmun Rev (2021) 20(3):102758. doi: 10.1016/j.autrev.2021.102758 33476818

[64] Ambarus CA Noordenbos T de Hair MJ Tak PP Baeten DL. Intimal Lining Layer Macrophages But Not Synovial Sublining Macrophages Display an IL-10 Polarized-Like Phenotype in Chronic Synovitis. Arthritis Res Ther (2012) 14(2):R74. doi: 10.1186/ar3796 22494514 PMC3446447

[65] Maruotti N Annese T Cantatore FP Ribatti D. Macrophages and Angiogenesis in Rheumatic Diseases. Vasc Cell (2013) 5(1):11. doi: 10.1186/2045-824X-5-11 23725043 PMC3680215

[66] Zhou M Qin S Chu Y Wang F Chen L Lu Y. Immunolocalization of MMP-2 and MMP-9 in Human Rheumatoid Synovium. Int J Clin Exp Pathol (2014) 7(6):3048–56. PMC4097284 25031723

[67] Suzuki S Morimoto S Fujishiro M Kawasaki M Hayakawa K Miyashita T. Inhibition of the Insulin-Like Growth Factor System is a Potential Therapy for Rheumatoid Arthritis. Autoimmunity (2015) 48(4):251–8. doi: 10.3109/08916934.2014.976631 25352179

[68] Henstridge CM Hyman BT Spires-Jones TL. Beyond the Neuron-Cellular Interactions Early in Alzheimer Disease Pathogenesis. Nat Rev Neurosci (2019) 20(2):94–108. doi: 10.1038/s41583-018-0113-1 30643230 PMC6545070

[69] Bhatia V Sharma S. Role of Mitochondrial Dysfunction, Oxidative Stress and Autophagy in Progression of Alzheimer’s Disease. J Neurol Sci (2021) 421:117253–75. doi: 10.1016/j.jns.2020.117253 33476985

[70] Galasko D Montine TJ. Biomarkers of Oxidative Damage and Inflammation in Alzheimer’s Disease. Biomark Med (2010) 4(1):27–36. doi: 10.2217/bmm.09.89 20383271 PMC2850111

[71] Thurgur H Pinteaux E. Microglia in the Neurovascular Unit: Blood-Brain Barrier-Microglia Interactions After Central Nervous System Disorders. Neuroscience (2019) 405:55–67. doi: 10.1016/j.neuroscience.2018.06.046 31007172

[72] Ebrahimpur M Sharifi F Shadman Z Payab M Mehraban S Shafiee G. Osteoporosis and Cognitive Impairment Interwoven Warning Signs: Community-Based Study on Older Adults-Bushehr Elderly Health (BEH) Program. Arch Osteoporos (2020) 15(1):140. doi: 10.1007/s11657-020-00817-1 32910343

[73] Amouzougan A Lafaie L Marotte H Dẻnariẻ D Collet P Pallot-Prades B. High Prevalence of Dementia in Women With Osteoporosis. Joint Bone Spine (2017) 84(5):611–4. doi: 10.1016/j.jbspin.2016.08.002 27697401

[74] Liu D Zhou H Tao Y Tan J Chen L Huang H. Alzheimer’s Disease is Associated With Increased Risk of Osteoporosis: The Chongqing Aging Study. Curr Alzheimer Res (2016) 13(10):1165–72. doi: 10.2174/15672050113109990149 23919776

[75] Davies DS Ma J Jegathees T Goldsbury C. Microglia Show Altered Morphology and Reduced Arborization in Human Brain During Aging and Alzheimer’s Disease. Brain Pathol (2017) 27(6):795–808. doi: 10.1111/bpa.12456 27862631 PMC8029278

[76] Xiang Z Haroutunian V Ho L Purohit D Pasinetti GM. Microglia Activation in the Brain as Inflammatory Biomarker of Alzheimer’s Disease Neuropathology and Clinical Dementia. Dis Markers (2006) 22(1-2):95–102. doi: 10.1155/2006/276239 16410654 PMC3850819

[77] Tang Y Le W. Differential Roles of M1 and M2 Microglia in Neurodegenerative Diseases. Mol Neurobiol (2016) 53(2):1181–94. doi: 10.1007/s12035-014-9070-5 25598354

[78] Sarlus H Heneka MT. Microglia in Alzheimer’s Disease. J Clin Invest (2017) 127(9):3240–9. doi: 10.1172/JCI90606 PMC5669553 28862638

[79] Schwab C McGeer PL. Inflammatory Aspects of Alzheimer Disease and Other Neurodegenerative Disorders. J Alzheimers Dis (2008) 13(4):359–69. doi: 10.3233/jad-2008-13402 18487845

[80] Luo XG Chen SD. The Changing Phenotype of Microglia From Homeostasis to Disease. Transl Neurodegener (2012) 1(1):9. doi: 10.1186/2047-9158-1-9 23210447 PMC3514090

[81] Varnum MM Ikezu T. The Classification of Microglial Activation Phenotypes on Neurodegeneration and Regeneration in Alzheimer’s Disease Brain. Arch Immunol Ther Exp (Warsz) (2012) 60(4):251–66. doi: 10.1007/s00005-012-0181-2 PMC4429536 22710659

[82] Wilkinson BL Landreth GE. The Microglial NADPH Oxidase Complex as a Source of Oxidative Stress in Alzheimer’s Disease. J Neuroinflamm (2006) 3:30–41. doi: 10.1186/1742-2094-3-30 PMC1637099 17094809

[83] Wang WY Tan MS Yu JT Tan L. Role of Pro-Inflammatory Cytokines Released From Microglia in Alzheimer’s Disease. Ann Transl Med (2015) 3(10):136. doi: 10.3978/j.issn.2305-5839.2015.03.49 26207229 PMC4486922

[84] Kaur D Sharma V Deshmukh R. Activation of Microglia and Astrocytes: A Roadway to Neuroinflammation and Alzheimer’s Disease. Inflammopharmacology (2019) 27(4):663–77. doi: 10.1007/s10787-019-00580-x 30874945

[85] Alves S Churlaud G Audrain M Michaelsen-Preusse K Fol R Souchet B. Interleukin-2 Improves Amyloid Pathology, Synaptic Failure and Memory in Alzheimer’s Disease Mice. Brain (2017) 140(3):826–42. doi: 10.1093/brain/aww330 28003243

[86] Ashutosh KW Cotter R Borgmann K Wu L Persidsky R. CXCL8 Protects Human Neurons From Amyloid-β-Induced Neurotoxicity: Relevance to Alzheimer’s Disease. Biochem Biophys Res Commun (2011) 412(4):565–71. doi: 10.1016/j.bbrc.2011.07.127 PMC3236067 21840299

[87] Gasparini L Xu H. Potential Roles of Insulin and IGF-1 in Alzheimer’s Disease. Trends Neurosci (2003) 26(8):404–6. doi: 10.1016/S0166-2236(03)00163-2 12900169

[88] Caruso G Fresta CG Musso N Giambirtone M Grasso M Spampinato SF. Carnosine Prevents Aβ-Induced Oxidative Stress and Inflammation in Microglial Cells: A Key Role of TGF-β1. Cells (2019) 8(1):64–86. doi: 10.3390/cells8010064 PMC6356400 30658430

[89] Ogunmokun G Dewanjee S Chakraborty P Valupadas C Chaudhary A Kolli V. The Potential Role of Cytokines and Growth Factors in the Pathogenesis of Alzheimer’s Disease. Cells (2021) 10(10):2790–816. doi: 10.3390/cells10102790 PMC8534363 34685770

[90] Durmanova V Javor J Parnicka Z Minarik G Ocenasova A Vaseckova B. Impact of MMP2 Rs243865 and MMP3 Rs3025058 Polymorphisms on Clinical Findings in Alzheimer’s Disease Patients. Mediat Inflamm (2021) 2021:5573642. doi: 10.1155/2021/5573642 PMC8079184 33986628

[91] Wang XX Tan MS Yu JT Tan L. Matrix Metalloproteinases and Their Multiple Roles in Alzheimer’s Disease. BioMed Res Int (2014) 2014:908636–43. doi: 10.1155/2014/908636 PMC4094696 25050378

[92] Hsieh TP Sheu SY Sun JS Chen MH. Icariin Inhibits Osteoclast Differentiation and Bone Resorption by Suppression of MAPKs/NF-κb Regulated HIF-1α and PGE(2) Synthesis. Phytomedicine (2011) 18(2-3):176–85. doi: 10.1016/j.phymed.2010.04.003 20554188

[93] Zhang D Zhang J Fong C Yao X Yang M. Herba Epimedii Flavonoids Suppress Osteoclastic Differentiation and Bone Resorption by Inducing G2/M Arrest and Apoptosis. Biochimie (2012) 94(12):2514–22. doi: 10.1016/j.biochi.2012.06.033 22796380

[94] Ma HP Ma XN Ge BF Zhen P Zhou J Gao YH. Icariin Attenuates Hypoxia-Induced Oxidative Stress and Apoptosis in Osteoblasts and Preserves Their Osteogenic Differentiation Potential *In Vitro* . Cell Prolif (2014) 47(6):527–39. doi: 10.1111/cpr.12147 PMC6496789 25355404

[95] Wu ZM Luo J Shi XD Zhang SX Zhu XB Guo J. Icariin Alleviates Rheumatoid Arthritis *via* Regulating miR-223-3p/NLRP3 Signalling Axis. Autoimmunity (2020) 53(8):450–8. doi: 10.1080/08916934.2020.1836488 33084415

[96] Liu T Zhao M Zhang Y Qiu Z Zhang Y Zhao C. Pharmacokinetic-Pharmacodynamic Modeling Analysis and Anti-Inflammatory Effect of Wangbi Capsule in the Treatment of Adjuvant-Induced Arthritis. BioMed Chromatogr (2021) 35(7):e5101. doi: 10.1002/bmc.5101 33625739

[97] Chi L Gao W Shu X Lu X. A Natural Flavonoid Glucoside, Icariin, Regulates Th17 and Alleviates Rheumatoid Arthritis in a Murine Model. Mediat Inflamm (2014) 2014:392062. doi: 10.1155/2014/392062 PMC4211316 25374443

[98] Luo H Zhang R. Icariin Enhances Cell Survival in Lipopolysaccharide-Induced Synoviocytes by Suppressing Ferroptosis *via* the Xc-/GPX4 Axis. Exp Ther Med (2021) 21(1):72. doi: 10.3892/etm.2020.9504 33365072 PMC7716635

[99] Choi EM. Modulatory Effects of Luteolin on Osteoblastic Function and Inflammatory Mediators in Osteoblastic MC3T3-E1 Cells. Cell Biol Int (2007) 31(9):870–7. doi: 10.1016/j.cellbi.2007.01.038 17368935

[100] Sun YW Bao Y Yu H Chen QJ Lu F Zhai S. Anti-Rheumatoid Arthritis Effects of Flavonoids From Daphne Genkwa. Int Immunopharmacol (2020) 83:106384. doi: 10.1016/j.intimp.2020.106384 32199350

[101] Abd EA Fathy MM Ali ZY El-Garawany A Mohamed EK. Enhanced Therapeutic Benefit of Quercetin-Loaded Phytosome Nanoparticles in Ovariectomized Rats. Chem Biol Interact (2017) 271:30–8. doi: 10.1016/j.cbi.2017.04.026 28460884

[102] Niu YB Yang YY Xiao X Sun Y Zhou YM Zhang YH. Quercetin Prevents Bone Loss in Hindlimb Suspension Mice *via* Stanniocalcin 1-Mediated Inhibition of Osteoclastogenesis. Acta Pharmacol Sin (2020) 41(11):1476–86. doi: 10.1038/s41401-020-00509-z PMC7656592 32934346

[103] Shen P Lin W Ba X Huang Y Chen Z Han L. Quercetin-Mediated SIRT1 Activation Attenuates Collagen-Induced Mice Arthritis. J Ethnopharmacol (2021) 279:114213. doi: 10.1016/j.jep.2021.114213 34023442

[104] Shangguan WJ Zhang YH Li ZC Tang LM Shao J Li H. Naringin Inhibits Vascular Endothelial Cell Apoptosis *via* Endoplasmic Reticulum Stress− and Mitochondrial−Mediated Pathways and Promotes Intraosseous Angiogenesis in Ovariectomized Rats. Int J Mol Med (2017) 40(6):1741–9. doi: 10.3892/ijmm.2017.3160 PMC5716435 29039439

[105] Cao X Lin W Liang C Zhang D Yang F Zhang Y. Naringin Rescued the TNF-α-Induced Inhibition of Osteogenesis of Bone Marrow-Derived Mesenchymal Stem Cells by Depressing the Activation of NF-Кb Signaling Pathway. Immunol Res (2015) 62(3):357–67. doi: 10.1007/s12026-015-8665-x 26032685

[106] Lee JH Kim GH. Evaluation of Antioxidant and Inhibitory Activities for Different Subclasses Flavonoids on Enzymes for Rheumatoid Arthritis. J Food Sci (2010) 75(7):H212–7. doi: 10.1111/j.1750-3841.2010.01755.x 21535545

[107] Aihaiti Y Song CY Tuerhong X Ni YY Ma Y Shi ZH. Therapeutic Effects of Naringin in Rheumatoid Arthritis: Network Pharmacology and Experimental Validation. Front Pharmacol (2021) 12:672054. doi: 10.3389/fphar.2021.672054 34054546 PMC8160516

[108] Zhai YK Guo XY Ge BF Zhen P Ma XN Zhou J. Icariin Stimulates the Osteogenic Differentiation of Rat Bone Marrow Stromal Cells *via* Activating the PI3K-AKT-eNOS-NO-cGMP-PKG. Bone (2014) 66:189–98. doi: 10.1016/j.bone.2014.06.016 24956021

[109] Hsieh TP Sheu SY Sun JS Chen MH Liu MH. Icariin Isolated From Epimedium Pubescens Regulates Osteoblasts Anabolism Through BMP-2, SMAD4, and Cbfa1 Expression. Phytomedicine (2010) 17(6):414–23. doi: 10.1016/j.phymed.2009.08.007 19747809

[110] Guo J Li F Wu Q Gong Q Lu Y Shi J. Protective Effects of Icariin on Brain Dysfunction Induced by Lipopolysaccharide in Rats. Phytomedicine (2010) 17(12):950–5. doi: 10.1016/j.phymed.2010.03.007 20382007

[111] Sze SC Tong Y Ng TB Cheng CL Cheung HP. Herba Epimedii: Anti-Oxidative Properties and Its Medical Implications. Molecules (2010) 15(11):7861–70. doi: 10.3390/molecules15117861 PMC6259234 21060294

[112] Luo Y Nie J Gong QH Lu YF Wu Q Shi JS. Protective Effects of Icariin Against Learning and Memory Deficits Induced by Aluminium in Rats. Clin Exp Pharmacol Physiol (2007) 34(8):792–5. doi: 10.1111/j.1440-1681.2007.04647.x 17600559

[113] Meng X Fu M Wang S Chen W Wang J Zhang N. Naringin Ameliorates Memory Deficits and Exerts Neuroprotective Effects in a Mouse Model of Alzheimer’s Disease by Regulating Multiple Metabolic Pathways. Mol Med Rep (2021) 23(5):332–44. doi: 10.3892/mmr.2021.11971 PMC7974313 33760152

[114] Sachdeva AK Kuhad A Chopra K. Naringin Ameliorates Memory Deficits in Experimental Paradigm of Alzheimer’s Disease by Attenuating Mitochondrial Dysfunction. Pharmacol Biochem Behav (2014) 127:101–10. doi: 10.1016/j.pbb.2014.11.002 25449356

[115] Wong SK Chin KY Ima-Nirwana S. Quercetin as an Agent for Protecting the Bone: A Review of the Current Evidence. Int J Mol Sci (2020) 21(17):6448–84. doi: 10.3390/ijms21176448 PMC7503351 32899435

[116] Yuan Z Min J Zhao Y Cheng Q Wang K Lin S. Quercetin Rescued TNF-Alpha-Induced Impairments in Bone Marrow-Derived Mesenchymal Stem Cell Osteogenesis and Improved Osteoporosis in Rats. Am J Transl Res (2018) 10(12):4313–21. PMC6325508 30662673

[117] Lu J Wu DM Zheng YL Hu B Zhang ZF Shan Q. Quercetin Activates AMP-Activated Protein Kinase by Reducing PP2C Expression Protecting Old Mouse Brain Against High Cholesterol-Induced Neurotoxicity. J Pathol (2010) 222(2):199–212. doi: 10.1002/path.2754 20690163

[118] Olayinka J Eduviere A Adeoluwa O Fafure A Adebanjo A Ozolua R. Quercetin Mitigates Memory Deficits in Scopolamine Mice Model *via* Protection Against Neuroinflammation and Neurodegeneration. Life Sci (2022) 292:120326. doi: 10.1016/j.lfs.2022.120326 35031260

