# Supplementary material for: Roles of Altered Macrophages and Cytokines: Implications for Pathological Mechanisms of Postmenopausal Osteoporosis, Rheumatoid Arthritis, and Alzheimer’s Disease
Source: Front Endocrinol (Lausanne). 2022 Jun 10;13:876269. doi: 10.3389/fendo.2022.876269 (PMC9226340; doi:10.3389/fendo.2022.876269)
Supplement: Supplementary file 1 [file DataSheet_1.docx]

**Table S1**. Summary of cytokines secreted by bone-bone marrow macrophages

| **Classification** | **Cytokines** | **Refs** |
| --- | --- | --- |
| Interleukin (IL) | IL-1, IL-2, IL-4, IL-6, IL-8, IL-10, IL-12, IL-13, IL-18, IL-23, IL-27 | (1-3) |
| Tumor necrosis factor (TNF) | TNF-α, TNF superfamily member 14 (TNFSF14/LIGHT) | (4, 5) |
| Interferon (IFN) | IFN-γ | (6) |
| Chemokines | CC motif chemokine ligand (CCL)1, CCL2, CCL5, C-X-C motif chemokine ligand (CXCL)9, CXCL10, CXCL11, CXCL12, CXCL13, CXCL16 | (2, 7, 8) |
| Others | Transforming growth factor-β (TGF-β), Bone morphogenetic protein-2 (BMP-2), BMP-4, BMP-6, Osteopontin (OPN) | (9, 10) |
|  | High mobility group box 1 (HMGB1), Chitinase 3 like 1 (CHI3L1/YKL-40), Oncostatin M (OSM), Prostaglandin E2 (PGE2), Cyclooxygenase-2 (COX-2), Insulin like growth factor 1 (IGF1) | (11-15) |
|  | Nitric oxide (NO), Inducible nitric oxide synthase (iNOS), Reactive oxygen species (ROS) | (4, 16) |
|  | Vascular endothelial growth factor (VEGF), Erythropoietin (EPO), Platelet derived growth factor-BB (PDGF-BB), Matrix metallopeptidase 2 (MMP2), MMP9, Fibroblast growth factor 2 (FGF2) | (2, 7, 17-19) |

**Table S2**. Effects of bone regulatory factors secreted by bone-bone marrow macrophages on bone formation and bone resorption

| **Cytokines** | **Main biological effects on coupling of bone formation and bone resorption** | **Refs** |
| --- | --- | --- |
| TGF-β | Promotes bone regeneration; regulates the proliferation, differentiation and mineralization of osteoblasts; and stimulates osteoclastogenesis directly. | (20-22) |
| BMP-2  BMP-4  BMP-6 | Have cross-talk with SMAD, WNT, RUNX2, and PTH signals to elevate bone formation in an ovariectomized (OVX)-induced osteoporosis model or bone cell model. | (23-29) |
| OPN | Suppresses osteoblast responses to mechanical stress and cytokines, including HGF and PDGF, and induces the increased bone resorption during bone remodeling. | (30-32) |

**Table S3**. Effects of cytokines-mediated inflammatory responses on bone formation and bone resorption

| **Cytokines** | **Main biological effects on coupling of bone formation and bone resorption** | **Refs** | **Main biological effects on uncoupling of bone formation and bone resorption through inflammatory response** | **Refs** |
| --- | --- | --- | --- | --- |
| Interleukins:  IL-1  IL-2  IL-4  IL-6  IL-8  IL-10  IL-12  IL-13  IL-18  IL-23  IL-27 | Pro-inflammatory cytokines, such as IL-1, IL-6, IL-8, and IL-23, promote osteoclasts activity, whereas anti-inflammatory cytokines such as IL-2, IL-4, IL-10, IL-12, IL-13, IL-18, and IL-27, inhibit proliferation and differentiation of osteoclasts. | (33-36) | Interleukins participate in differentiation of T cells into Th-17 cells (such as IL-1, IL-6, and IL-23), multinucleated giant cells formation (such as IL-4 and IL-13), and secretion of cytokines, thus regulating osteoclast formation and differentiation. | (6, 37-39) |
| TNF-α  TNFSF14 (LIGHT) | Regulate osteoclast formation indirectly or directly through the OPG/RANK/RANKL system. | (40-42) | TNF promotes osteoclast formation by switching the differentiation of M-CSF-induced M2 residents to M1 inflammatory macrophages and acting synergistically with IL-1. | (43, 44) |
| IFN-γ | Stimulates bone marrow mesenchymal stem cells to differentiate into osteoblasts; inhibits bone marrow adipocyte formation; and plays a dual role in osteoclasts depending on their stage. | (45, 46) | Promoted the expression of CIITA in immune cells to enhance the antigen presentation between macrophages and T cells, then up-regulated TNF-α and RANKL and promoted bone resorption. | (47, 48) |
| Chemokines:  CCL2  CCL5  CXCL9  CXCL10  CXCL11  CXCL12  CXCL13  CXCL16 | Chemokines are associated with osteoclastogenesis include CCL2, CXCL10, CXCL11, and CXCL16, while CCL5, CXCL9 (negative regulator), CXCL12, and CXCL13 are associated with bone formation. | (33, 49-55) | Chemokines (such as CCL2, CCL5, and CXCL10) and elevated levels of a wide range of proinflammatory cytokines (IL-1, IL-6, IL-15, IL-17, IL-18, IL-21, IL-22, IL-23, and TNF-α) act in synergy with M-CSF/RANK signals to promote osteoclastogenesis. | (33, 56) |
| HMGB1 | Participates in RANKL-induced and integrin-dependent osteoclastogenesis, and acts as a chemotactic stimulus to osteoclasts. | (57, 58) | Stimulates the expression of TNF-α and IL-6 cytokines, further enhances inflammatory bone loss. | (58, 59) |
| YKL-40 (CHI3L1) | Silencing YKL-40 resulted in a significant decrease in bone resorption activity. | (60) | A new pro-inflammatory biomarker that may initiate or stimulate inflammatory processes and indirectly contribute to bone destruction. | (61) |
| OSM | A key monocyte-derived osteogenic factor that promotes osteogenic differentiation via gp130 and STAT3 signaling pathways, and inhibits bone resorption. | (62-64) | Induces cells in the osteoblastic lineage to secrete IL-6. | (64) |
| PGE2 | Promotes osteogenic differentiation directly, and bone formation by inducing OSM and BMP-2 production in macrophages. | (65-67) | Involved in bone resorption elicited by lipopolysaccharide. | (68) |
| COX-2 | In knockout mice, osteogenesis was impaired and bone metabolic turnover was reduced significantly. | (69, 70) | Responsible for the conversion of arachidonic acid to prostaglandins (such as PGE2) during the early inflammatory phase of bone repair. | (71, 72) |
| IGF-1 | Reduced bone mineral content and stimulated bone resorption in older OVX rats. | (73) | Participates in the immunomodulation  of osteogenesis during transition of M1/M2 macrophages in vitro. | (74) |

**Table S4**. Effects of cytokines-mediated oxidative stress on bone formation and bone resorption

| **Cytokines** | **Main biological effects on coupling of bone formation and bone resorption** | **Refs** | **Main biological effects on uncoupling of bone formation and bone resorption through oxidative stress** | **Refs** |
| --- | --- | --- | --- | --- |
| NO  iNOS | Involved in the regulation of osteoclast formation and differentiation. | (75) | Reacts with superoxide anion to produce peroxynitrite, which appears to be directly involved in both bone degradation and osteoclast formation. | (76, 77) |
| ROS | Promotes the apoptosis of osteoblasts and osteoclasts, and enhances the activity, proliferation, and differentiation of osteoclasts. | (78, 79) | Elimination of ROS generation alleviates OVX-induced oxidative stress and inhibits bone resorption through RANKL-mediated NF-κB activation. | (80, 81) |

**Table S5**. Effects of cytokines-mediated angiogenesis on bone formation and bone resorption

| **Cytokines** | **Main biological effects on coupling of bone formation and bone resorption** | **Refs** | **Main biological effects on the uncoupling of bone formation and bone resorption through angiogenesis** | **Refs** |
| --- | --- | --- | --- | --- |
| VEGF | Regulates osteoblast differentiation and mineralization, and promotes the release of osteogenic factors from blood vessels. | (82-84) | Increased the number of vessels and changed the composition of the vascular supply. | (85, 86) |
| EPO | Maintains the balance between osteogenesis and adipogenesis in the bone marrow, and inhibits bone resorption. | (87, 88) | Induced angiogenic differentiation of human endothelial cells and mesenchymal stem cells, and promoted endosteal vascularization in a model of fracture repair. | (89, 90) |
| PDGF-BB | Promotes the recruitment of endothelial progenitor cells and mesenchymal stem cells, and repair of the skeletal system. | (91-93) | Induced the formation of type H vessels and restored bone mass in a OVX mice. | (91, 94, 95) |
| MMP2 | Elevated in OVX rats and degraded type I collagen barrier bone cells, subsequently in active osteoclasts. | (96, 97) | Reduced angiogenesis of human vascular  endothelial cells via the HIF-1α signaling pathway, which ultimately accelerated bone formation. | (98) |
| MMP9 | Up-regulated during osteoclast differentiation, and promoted bone resorption. | (99, 100) | Increased vascularization during bone formation and  promoted angiogenesis  in response to PTH-related peptide or RANKL in explants. | (101, 102) |
| FGF2 | Stimulates osteoblast precursor proliferation, thereby promoting bone formation. | (103, 104) | Stimulated the proliferation of endothelial cells and secretion of MMPs by inducing secretion of VEGF. | (105) |

**METHODS OF BIOINFORMATICS**

**Differential Gene Screening of Postmenopausal Osteoporosis (PMOP), Rheumatoid Arthritis (RA), and Alzheimer's Disease (AD)**

Differential genes of PMOP, RA, and AD were obtained by GEO, DisGeNET, DrugBank, GeneCards, MalaCards, OMIM, Pharmgkb, Phenopedia, TTD, and KEGG Disease databases, respectively. (106-115). The original file of three chips, including GSE56116, GSE17755, and GSE48350, screened on the GEO database, was processed by a robust multiarray average algorithm with normalization of matrix data. The relevant data were filtered using the Limma package to analyze the chip data twice, combining the *p*-value and the difference multiple. The screening conditions for significantly differentially expressed genes were *p* < 0.05 with a |log 2 (fold change)| > 0.05. The screening results of different genes in all databases are shown in Table S6. Then, the differential genes screened by multiple data sets were merged, and the duplicate genes were removed. The final genes are the total differential genes for each disease.

**Enrichment Analysis of Kyoto Encyclopedia of Genes and Genomes (KEGG) Pathway of Differential Genes in PMOP**

The KEGG database (https://www.kegg.jp/) was used to identify the function and biological correlation of candidate target genes (116). The Cluster Profiler R package was used to visualize the KEGG pathway data. The pathways that exhibited significant changes with a false discovery rate (FDR) < 0.05 were selected for further analysis, and the top 20 KEGG pathways were selected.

**Gene Ontology (GO) Functional Enrichment Analysis Was Performed on the Intersection Difference Genes of PMOP vs. RA and PMOP vs. AD**

The Gene Ontology database (GO, http://geneontology.org/) was used to identify biological mechanisms from high-throughput genomic or transcriptome data (117). The Cluster Profiler R package was used to visualize the GO function data. The functional categories were enriched within genes (false discovery rate [FDR] < 0.05), and the top 20 GO functional categories were selected.

**Construction of the Core Network of Protein-Protein Interaction (PPI) and Analysis of Biological Processes in the Common Pathological Molecules of PMOP vs. RA and PMOP vs. AD**

The intersection genes of PMOP and RA, as well as PMOP and AD, were respectively used to construct the PPI network through the STRING 11.0 database (https://www.string-db.org/) (highest confidence > 0.9) (118). Then, using the network topology analysis plugin CytoNCA and filtering with Betweenness Centrality (BC), Degree Centrality (DC), Closer Centrality (CC), Eigenvector Centrality (EC), the Local Average Connectivity-Based Method (LAC), and Network Centrality (NC), key genes were identified in the PPI network. Finally, the core genes were imported into the STRING database, and the biological process was analyzed.

**Table S6**. Summary of PMOP, RA, and AD differential genes

| **Database** | **Number of differential genes** | | | **Website** |
| --- | --- | --- | --- | --- |
|  | **PMOP** | **RA** | **AD** |  |
| GEO | 971  (GSE56116) | 47  (GSE17755) | 78  (GSE48350) | https://www.ncbi.nlm.nih.gov/ |
| Disgenet | 171 | 2090 | 3485 | http://www.disgenet.org/ |
| DrugBank | 103 | 94 | 90 | https://www.drugbank.com/ |
| GeneCards  (score≥1) | 896 | 1868 | 8773 | https://www.genecards.org/ |
| MalaCards | 0 | 471 | 569 | http://www.malacards.org/ |
| OMIM | 10 | 25 | 143 | https://omim.org/ |
| Pharmgkb | 15 | 9 | 87 | https://www.pharmgkb.org/ |
| Phenopedia | 4 | 251 | 834 | https://phgkb.cdc.gov/PHGKB/startP-agePhenoPedia.action |
| TTD | 4 | 93 | 132 | http://db.idrblab.net/ttd/ |
| KEGG | 0 | 19 | 0 | <https://www.kegg.jp/kegg/genes.>html |


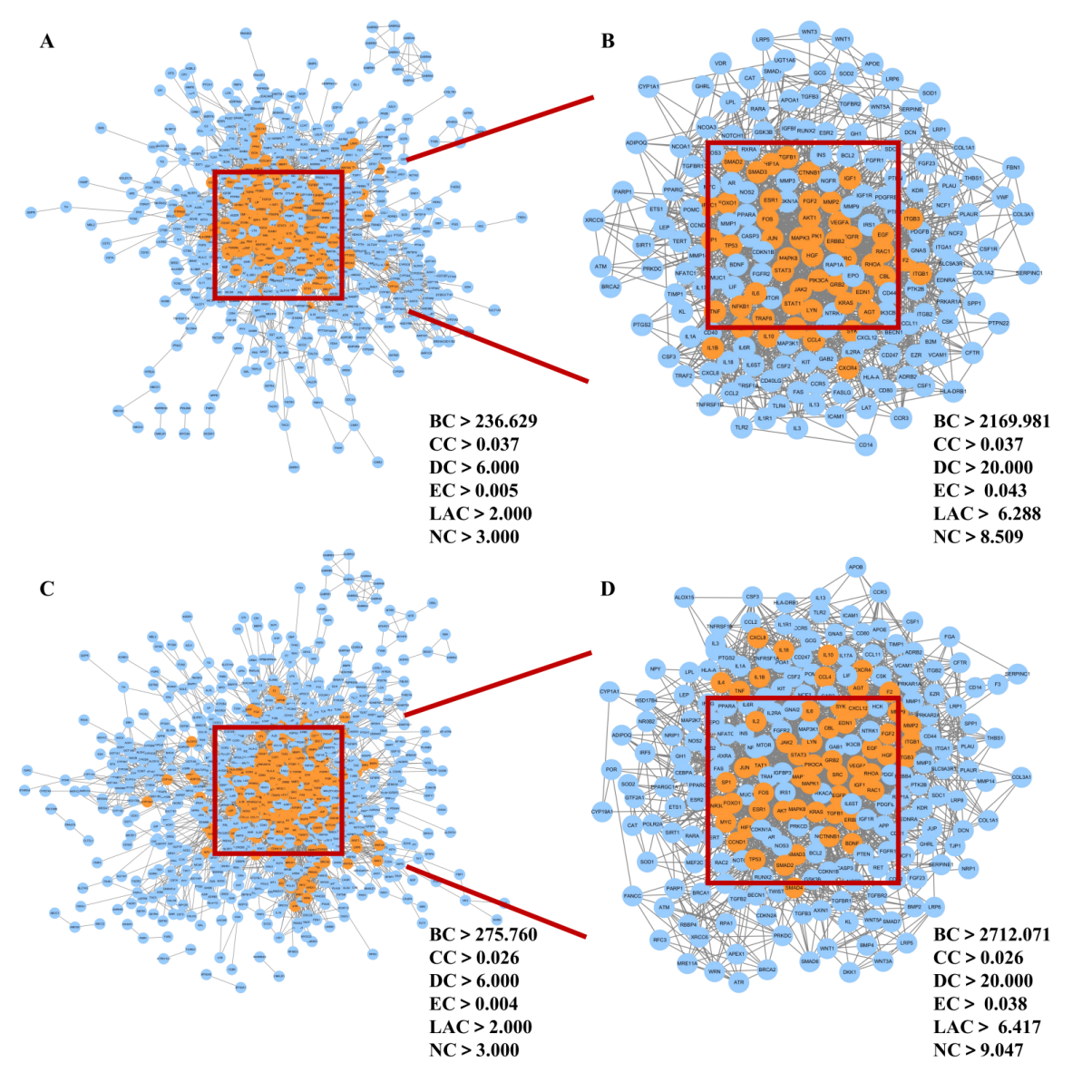


**Figure S1**. Protein-protein interaction (PPI) network topology analysis was performed for common differential genes in PMOP vs. RA (**A, B**) and PMOP vs. AD (**C, D**). The intersection targets shown in the PPI network were performed twice for topology network analysis by CytoNCA. Parameters such as BC, CC, DC, EC, LAC, and NC represent the filter conditions. The orange nodes represent the key targets in the subnetwork obtained from topology analysis; every node with a score higher than the median will be selected; and the final key subnetwork will be screened after two filtrations.

**REFERENCES**

1. Chen K, Jiao Y, Liu L, Huang M, He C, He W, et al. Communications Between Bone Marrow Macrophages and Bone Cells in Bone Remodeling. Front Cell Dev Biol. [Journal Article; Review]. 2020 2020-01-20;8:598263.

2. Muñoz J, Akhavan NS, Mullins AP, Arjmandi BH. Macrophage Polarization and Osteoporosis: A Review. NUTRIENTS. [Journal Article; Review]. 2020 2020-09-30;12(10).

3. Suzuki E, Sugiyama C, Umezawa K. Inhibition of inflammatory mediator secretion by (-)-DHMEQ in mouse bone marrow-derived macrophages. BIOMED PHARMACOTHER. [Journal Article]. 2009 2009-06-01;63(5):351-8.

4. Chen M, Zhang Y, Zhou P, Liu X, Zhao H, Zhou X, et al. Substrate stiffness modulates bone marrow-derived macrophage polarization through NF-κB signaling pathway. Bioact Mater. [Journal Article]. 2020 2020-12-01;5(4):880-90.

5. Edwards JP, Zhang X, Frauwirth KA, Mosser DM. Biochemical and functional characterization of three activated macrophage populations. J Leukoc Biol. [Comparative Study; Journal Article; Research Support, N.I.H., Extramural]. 2006 2006-12-01;80(6):1298-307.

6. Yang DH, Yang MY. The Role of Macrophage in the Pathogenesis of Osteoporosis. INT J MOL SCI. [Journal Article; Review]. 2019 2019-04-28;20(9).

7. Zhao H, Kalish FS, Wong RJ, Stevenson DK. Hypoxia regulates placental angiogenesis via alternatively activated macrophages. AM J REPROD IMMUNOL. [Journal Article; Research Support, Non-U.S. Gov't]. 2018 2018-09-01;80(3):e12989.

8. Fleetwood AJ, Dinh H, Cook AD, Hertzog PJ, Hamilton JA. GM-CSF- and M-CSF-dependent macrophage phenotypes display differential dependence on type I interferon signaling. J Leukoc Biol. [Comparative Study; Journal Article; Research Support, Non-U.S. Gov't]. 2009 2009-08-01;86(2):411-21.

9. Michalski MN, McCauley LK. Macrophages and skeletal health. Pharmacol Ther. [Journal Article; Review]. 2017 2017-06-01;174:43-54.

10. Anisiewicz A, Łabędź N, Krauze I, Wietrzyk J. Calcitriol in the Presence of Conditioned Media from Metastatic Breast Cancer Cells Enhances Ex Vivo Polarization of M2 Alternative Murine Bone Marrow-Derived Macrophages. Cancers (Basel). [Journal Article]. 2020 2020-11-23;12(11).

11. Andersson A, Covacu R, Sunnemark D, Danilov AI, Dal Bianco A, Khademi M, et al. Pivotal advance: HMGB1 expression in active lesions of human and experimental multiple sclerosis. J Leukoc Biol. [Journal Article; Research Support, Non-U.S. Gov't]. 2008 2008-11-01;84(5):1248-55.

12. Yang PS, Yu MH, Hou YC, Chang CP, Lin SC, Kuo IY, et al. Targeting protumor factor chitinase-3-like-1 secreted by Rab37 vesicles for cancer immunotherapy. THERANOSTICS. [Journal Article; Research Support, Non-U.S. Gov't]. 2022 2022-01-20;12(1):340-61.

13. Guihard P, Boutet MA, Brounais-Le RB, Gamblin AL, Amiaud J, Renaud A, et al. Oncostatin m, an inflammatory cytokine produced by macrophages, supports intramembranous bone healing in a mouse model of tibia injury. AM J PATHOL. [Journal Article; Research Support, Non-U.S. Gov't]. 2015 2015-03-01;185(3):765-75.

14. Estus TL, Choudhary S, Pilbeam CC. Prostaglandin-mediated inhibition of PTH-stimulated β-catenin signaling in osteoblasts by bone marrow macrophages. BONE. [Journal Article; Research Support, N.I.H., Extramural]. 2016 2016-04-01;85:123-30.

15. Barrett JP, Minogue AM, Falvey A, Lynch MA. Involvement of IGF-1 and Akt in M1/M2 activation state in bone marrow-derived macrophages. EXP CELL RES. [Journal Article; Research Support, Non-U.S. Gov't]. 2015 2015-07-15;335(2):258-68.

16. Kim SJ, Ko WK, Jo MJ, Arai Y, Choi H, Kumar H, et al. Anti-inflammatory effect of Tauroursodeoxycholic acid in RAW 264.7 macrophages, Bone marrow-derived macrophages, BV2 microglial cells, and spinal cord injury. Sci Rep. [Journal Article; Research Support, Non-U.S. Gov't]. 2018 2018-02-16;8(1):3176.

17. Kaur S, Raggatt LJ, Batoon L, Hume DA, Levesque JP, Pettit AR. Role of bone marrow macrophages in controlling homeostasis and repair in bone and bone marrow niches. SEMIN CELL DEV BIOL. [Journal Article; Research Support, Non-U.S. Gov't; Review]. 2017 2017-01-01;61:12-21.

18. Spiller KL, Wrona EA, Romero-Torres S, Pallotta I, Graney PL, Witherel CE, et al. Differential gene expression in human, murine, and cell line-derived macrophages upon polarization. EXP CELL RES. [Journal Article; Research Support, N.I.H., Extramural; Research Support, Non-U.S. Gov't]. 2016 2016-09-10;347(1):1-13.

19. Gao B, Deng R, Chai Y, Chen H, Hu B, Wang X, et al. Macrophage-lineage TRAP+ cells recruit periosteum-derived cells for periosteal osteogenesis and regeneration. J CLIN INVEST. [Journal Article; Research Support, N.I.H., Extramural]. 2019 2019-04-04;129(6):2578-94.

20. Aslani S, Abhari A, Sakhinia E, Sanajou D, Rajabi H, Rahimzadeh S. Interplay between microRNAs and Wnt, transforming growth factor-β, and bone morphogenic protein signaling pathways promote osteoblastic differentiation of mesenchymal stem cells. J CELL PHYSIOL. [Journal Article; Review]. 2019 2019-06-01;234(6):8082-93.

21. Mei L, Sang W, Chen Z, Zheng L, Jin K, Lou C, et al. Small molecule inhibitor RepSox prevented ovariectomy-induced osteoporosis by suppressing osteoclast differentiation and bone resorption. J CELL PHYSIOL. [Journal Article; Research Support, Non-U.S. Gov't]. 2018 2018-12-01;233(12):9724-38.

22. Zuo C, Huang Y, Bajis R, Sahih M, Li YP, Dai K, et al. Osteoblastogenesis regulation signals in bone remodeling. Osteoporos Int. [Journal Article; Research Support, Non-U.S. Gov't; Review]. 2012 2012-06-01;23(6):1653-63.

23. Chai S, Wan L, Wang JL, Huang JC, Huang HX. Gushukang inhibits osteocyte apoptosis and enhances BMP-2/Smads signaling pathway in ovariectomized rats. PHYTOMEDICINE. [Journal Article]. 2019 2019-11-01;64:153063.

24. Jian J, Sun L, Cheng X, Hu X, Liang J, Chen Y. Calycosin-7-O-β-d-glucopyranoside stimulates osteoblast differentiation through regulating the BMP/WNT signaling pathways. ACTA PHARM SIN B. [Journal Article]. 2015 2015-09-01;5(5):454-60.

25. Ponzetti M, Rucci N. Osteoblast Differentiation and Signaling: Established Concepts and Emerging Topics. INT J MOL SCI. [Journal Article; Review]. 2021 2021-06-22;22(13).

26. Khan MP, Khan K, Yadav PS, Singh AK, Nag A, Prasahar P, et al. BMP signaling is required for adult skeletal homeostasis and mediates bone anabolic action of parathyroid hormone. BONE. [Journal Article; Research Support, Non-U.S. Gov't]. 2016 2016-11-01;92:132-44.

27. Hsu SL, Chou WY, Hsu CC, Ko JY, Jhan SW, Wang CJ, et al. Shockwave Therapy Modulates the Expression of BMP2 for Prevention of Bone and Cartilage Loss in the Lower Limbs of Postmenopausal Osteoporosis Rat Model. Biomedicines. [Journal Article]. 2020 2020-12-15;8(12).

28. Lu XD, Han WX, Liu YX. Suppression of miR-451a accelerates osteogenic differentiation and inhibits bone loss via Bmp6 signaling during osteoporosis. BIOMED PHARMACOTHER. [Journal Article]. 2019 2019-12-01;120:109378.

29. Simic P, Culej JB, Orlic I, Grgurevic L, Draca N, Spaventi R, et al. Systemically administered bone morphogenetic protein-6 restores bone in aged ovariectomized rats by increasing bone formation and suppressing bone resorption. J BIOL CHEM. [Journal Article; Research Support, Non-U.S. Gov't]. 2006 2006-09-01;281(35):25509-21.

30. Kusuyama J, Bandow K, Ohnishi T, Hisadome M, Shima K, Semba I, et al. Osteopontin inhibits osteoblast responsiveness through the down-regulation of focal adhesion kinase mediated by the induction of low-molecular weight protein tyrosine phosphatase. MOL BIOL CELL. [Journal Article]. 2017 2017-05-15;28(10):1326-36.

31. Malaval L, Wade-Guéye NM, Boudiffa M, Fei J, Zirngibl R, Chen F, et al. Bone sialoprotein plays a functional role in bone formation and osteoclastogenesis. J EXP MED. [Journal Article; Research Support, Non-U.S. Gov't]. 2008 2008-05-12;205(5):1145-53.

32. Chang IC, Chiang TI, Yeh KT, Lee H, Cheng YW. Increased serum osteopontin is a risk factor for osteoporosis in menopausal women. Osteoporos Int. [Journal Article; Research Support, Non-U.S. Gov't]. 2010 2010-08-01;21(8):1401-9.

33. Amarasekara DS, Yun H, Kim S, Lee N, Kim H, Rho J. Regulation of Osteoclast Differentiation by Cytokine Networks. IMMUNE NETW. [Journal Article; Review]. 2018 2018-02-01;18(1):e8.

34. Lapérine O, Blin-Wakkach C, Guicheux J, Beck-Cormier S, Lesclous P. Dendritic-cell-derived osteoclasts: a new game changer in bone-resorption-associated diseases. DRUG DISCOV TODAY. [Journal Article; Research Support, Non-U.S. Gov't; Review]. 2016 2016-09-01;21(9):1345-54.

35. Lee SK, Lorenzo J. Cytokines regulating osteoclast formation and function. CURR OPIN RHEUMATOL. [Journal Article; Review]. 2006 2006-07-01;18(4):411-8.

36. Datta HK, Ng WF, Walker JA, Tuck SP, Varanasi SS. The cell biology of bone metabolism. J CLIN PATHOL. [Journal Article; Review]. 2008 2008-05-01;61(5):577-87.

37. Wang T, He C. TNF-α and IL-6: The Link between Immune and Bone System. CURR DRUG TARGETS. [Journal Article; Research Support, Non-U.S. Gov't; Review]. 2020 2020-01-20;21(3):213-27.

38. Pagliari D, Ciro TF, Zirio G, Newton EE, Cianci R. The role of "bone immunological niche" for a new pathogenetic paradigm of osteoporosis. Anal Cell Pathol (Amst). [Journal Article; Review]. 2015 2015-01-20;2015:434389.

39. Li X, Luo W, Hu J, Chen Y, Yu T, Yang J, et al. Interleukin-27 prevents LPS-induced inflammatory osteolysis by inhibiting osteoclast formation and function. AM J TRANSL RES. [Journal Article; Review]. 2019 2019-01-20;11(3):1154-69.

40. Marahleh A, Kitaura H, Ohori F, Kishikawa A, Ogawa S, Shen WR, et al. TNF-α Directly Enhances Osteocyte RANKL Expression and Promotes Osteoclast Formation. FRONT IMMUNOL. [Journal Article; Research Support, Non-U.S. Gov't]. 2019 2019-01-20;10:2925.

41. Vural P, Canbaz M, Akgul C. Effects of menopause and postmenopausal tibolone treatment on plasma TNFalpha, IL-4, IL-10, IL-12 cytokine pattern and some bone turnover markers. PHARMACOL RES. [Journal Article]. 2006 2006-04-01;53(4):367-71.

42. Brunetti G, Faienza MF, Colaianni G, Gigante I, Oranger A, Pignataro P, et al. Impairment of Bone Remodeling in LIGHT/TNFSF14-Deficient Mice. J BONE MINER RES. [Journal Article; Research Support, N.I.H., Extramural; Research Support, Non-U.S. Gov't]. 2018 2018-04-01;33(4):704-19.

43. Zhao Z, Hou X, Yin X, Li Y, Duan R, Boyce BF, et al. TNF Induction of NF-κB RelB Enhances RANKL-Induced Osteoclastogenesis by Promoting Inflammatory Macrophage Differentiation but also Limits It through Suppression of NFATc1 Expression. PLOS ONE. [Journal Article; Research Support, N.I.H., Extramural; Research Support, Non-U.S. Gov't]. 2015 2015-01-20;10(8):e135728.

44. Yao Z, Xing L, Qin C, Schwarz EM, Boyce BF. Osteoclast precursor interaction with bone matrix induces osteoclast formation directly by an interleukin-1-mediated autocrine mechanism. J BIOL CHEM. [Journal Article; Research Support, N.I.H., Extramural]. 2008 2008-04-11;283(15):9917-24.

45. Tang M, Tian L, Luo G, Yu X. Interferon-Gamma-Mediated Osteoimmunology. FRONT IMMUNOL. [Journal Article; Review]. 2018 2018-01-20;9:1508.

46. Xiong Q, Zhang L, Ge W, Tang P. The roles of interferons in osteoclasts and osteoclastogenesis. JOINT BONE SPINE. [Journal Article; Review]. 2016 2016-05-01;83(3):276-81.

47. Roggia C, Tamone C, Cenci S, Pacifici R, Isaia GC. Role of TNF-alpha producing T-cells in bone loss induced by estrogen deficiency. MINERVA MED. [Journal Article]. 2004 2004-04-01;95(2):125-32.

48. Weitzmann MN, Pacifici R. Estrogen regulation of immune cell bone interactions. Ann N Y Acad Sci. [Journal Article; Review]. 2006 2006-04-01;1068:256-74.

49. Khan UA, Hashimi SM, Bakr MM, Forwood MR, Morrison NA. CCL2 and CCR2 are Essential for the Formation of Osteoclasts and Foreign Body Giant Cells. J CELL BIOCHEM. [Journal Article; Research Support, Non-U.S. Gov't]. 2016 2016-02-01;117(2):382-9.

50. Zhao Y, Jia L, Zheng Y, Li W. Involvement of Noncoding RNAs in the Differentiation of Osteoclasts. STEM CELLS INT. [Journal Article; Review]. 2020 2020-01-20;2020:4813140.

51. Li C, Zhao J, Sun L, Yao Z, Liu R, Huang J, et al. RANKL downregulates cell surface CXCR6 expression through JAK2/STAT3 signaling pathway during osteoclastogenesis. Biochem Biophys Res Commun. [Journal Article; Research Support, Non-U.S. Gov't]. 2012 2012-12-14;429(3-4):156-62.

52. Wintges K, Beil FT, Albers J, Jeschke A, Schweizer M, Claass B, et al. Impaired bone formation and increased osteoclastogenesis in mice lacking chemokine (C-C motif) ligand 5 (Ccl5). J BONE MINER RES. [Journal Article; Research Support, Non-U.S. Gov't]. 2013 2013-10-01;28(10):2070-80.

53. Huang B, Wang W, Li Q, Wang Z, Yan B, Zhang Z, et al. Osteoblasts secrete Cxcl9 to regulate angiogenesis in bone. NAT COMMUN. [Journal Article; Research Support, Non-U.S. Gov't]. 2016 2016-12-14;7:13885.

54. Higashino K, Viggeswarapu M, Bargouti M, Liu H, Titus L, Boden SD. Stromal cell-derived factor-1 potentiates bone morphogenetic protein-2 induced bone formation. Tissue Eng Part A. [Journal Article; Research Support, Non-U.S. Gov't; Research Support, U.S. Gov't, Non-P.H.S.]. 2011 2011-02-01;17(3-4):523-30.

55. Tian F, Ji XL, Xiao WA, Wang B, Wang F. CXCL13 Promotes Osteogenic Differentiation of Mesenchymal Stem Cells by Inhibiting miR-23a Expression. STEM CELLS INT. [Journal Article]. 2015 2015-01-20;2015:632305.

56. Sucur A, Jajic Z, Artukovic M, Matijasevic MI, Anic B, Flegar D, et al. Chemokine signals are crucial for enhanced homing and differentiation of circulating osteoclast progenitor cells. ARTHRITIS RES THER. [Journal Article; Research Support, Non-U.S. Gov't]. 2017 2017-06-15;19(1):142.

57. Zhou Z, Han JY, Xi CX, Xie JX, Feng X, Wang CY, et al. HMGB1 regulates RANKL-induced osteoclastogenesis in a manner dependent on RAGE. J BONE MINER RES. [Journal Article; Research Support, N.I.H., Extramural]. 2008 2008-07-01;23(7):1084-96.

58. De Martinis M, Ginaldi L, Sirufo MM, Pioggia G, Calapai G, Gangemi S, et al. Alarmins in Osteoporosis, RAGE, IL-1, and IL-33 Pathways: A Literature Review. Medicina (Kaunas). [Journal Article; Review]. 2020 2020-03-19;56(3).

59. Sapra L, Azam Z, Rani L, Saini C, Bhardwaj A, Shokeen N, et al. “Immunoporosis”: Immunology of Osteoporosis. Proceedings of the National Academy of Sciences, India Section B: Biological Sciences. 2021 2021-01-01;91(3):511-9.

60. Di Rosa M, Tibullo D, Vecchio M, Nunnari G, Saccone S, Di Raimondo F, et al. Determination of chitinases family during osteoclastogenesis. BONE. [Journal Article]. 2014 2014-04-01;61:55-63.

61. Steinke J, Samietz S, Friedrich N, Weiss S, Michalik S, Biffar R, et al. Associations of plasma YKL-40 concentrations with heel ultrasound parameters and bone turnover markers in the general adult population. BONE. [Journal Article; Research Support, Non-U.S. Gov't]. 2020 2020-12-01;141:115675.

62. Nicolaidou V, Wong MM, Redpath AN, Ersek A, Baban DF, Williams LM, et al. Monocytes induce STAT3 activation in human mesenchymal stem cells to promote osteoblast formation. PLOS ONE. [Journal Article; Research Support, Non-U.S. Gov't]. 2012 2012-01-20;7(7):e39871.

63. Malaval L, Liu F, Vernallis AB, Aubin JE. GP130/OSMR is the only LIF/IL-6 family receptor complex to promote osteoblast differentiation of calvaria progenitors. J CELL PHYSIOL. [Journal Article; Research Support, Non-U.S. Gov't]. 2005 2005-08-01;204(2):585-93.

64. Jay PR, Centrella M, Lorenzo J, Bruce AG, Horowitz MC. Oncostatin-M: a new bone active cytokine that activates osteoblasts and inhibits bone resorption. ENDOCRINOLOGY. [Journal Article; Research Support, U.S. Gov't, Non-P.H.S.; Research Support, U.S. Gov't, P.H.S.]. 1996 1996-04-01;137(4):1151-8.

65. Zhang M, Feigenson M, Sheu TJ, Awad HA, Schwarz EM, Jonason JH, et al. Loss of the PGE2 receptor EP1 enhances bone acquisition, which protects against age and ovariectomy-induced impairments in bone strength. BONE. [Journal Article; Research Support, N.I.H., Extramural; Research Support, Non-U.S. Gov't]. 2015 2015-03-01;72:92-100.

66. Guihard P, Danger Y, Brounais B, David E, Brion R, Delecrin J, et al. Induction of osteogenesis in mesenchymal stem cells by activated monocytes/macrophages depends on oncostatin M signaling. STEM CELLS. [Journal Article; Research Support, Non-U.S. Gov't]. 2012 2012-04-01;30(4):762-72.

67. Kanayama S, Kaito T, Kitaguchi K, Ishiguro H, Hashimoto K, Chijimatsu R, et al. ONO-1301 Enhances in vitro Osteoblast Differentiation and in vivo Bone Formation Induced by Bone Morphogenetic Protein. Spine (Phila Pa 1976). [Journal Article]. 2018 2018-06-01;43(11):E616-24.

68. Miyaura C, Inada M, Matsumoto C, Ohshiba T, Uozumi N, Shimizu T, et al. An essential role of cytosolic phospholipase A2alpha in prostaglandin E2-mediated bone resorption associated with inflammation. J EXP MED. [Journal Article; Research Support, Non-U.S. Gov't]. 2003 2003-05-19;197(10):1303-10.

69. Zhang X, Schwarz EM, Young DA, Puzas JE, Rosier RN, O'Keefe RJ. Cyclooxygenase-2 regulates mesenchymal cell differentiation into the osteoblast lineage and is critically involved in bone repair. J CLIN INVEST. [Journal Article; Research Support, Non-U.S. Gov't; Research Support, U.S. Gov't, P.H.S.]. 2002 2002-06-01;109(11):1405-15.

70. Tanaka Y, Nakayamada S, Okada Y. Osteoblasts and osteoclasts in bone remodeling and inflammation. Curr Drug Targets Inflamm Allergy. [Journal Article; Review]. 2005 2005-06-01;4(3):325-8.

71. Lu LY, Loi F, Nathan K, Lin TH, Pajarinen J, Gibon E, et al. Pro-inflammatory M1 macrophages promote Osteogenesis by mesenchymal stem cells via the COX-2-prostaglandin E2 pathway. J ORTHOP RES. [Journal Article]. 2017 2017-11-01;35(11):2378-85.

72. Blackwell KA, Raisz LG, Pilbeam CC. Prostaglandins in bone: bad cop, good cop? Trends Endocrinol Metab. [Journal Article; Research Support, N.I.H., Extramural; Review]. 2010 2010-05-01;21(5):294-301.

73. Ibbotson KJ, Orcutt CM, D'Souza SM, Paddock CL, Arthur JA, Jankowsky ML, et al. Contrasting effects of parathyroid hormone and insulin-like growth factor I in an aged ovariectomized rat model of postmenopausal osteoporosis. J BONE MINER RES. [Comparative Study; Journal Article]. 1992 1992-04-01;7(4):425-32.

74. Córdova LA, Loi F, Lin TH, Gibon E, Pajarinen J, Nabeshima A, et al. CCL2, CCL5, and IGF-1 participate in the immunomodulation of osteogenesis during M1/M2 transition in vitro. J BIOMED MATER RES A. [Journal Article]. 2017 2017-11-01;105(11):3069-76.

75. Yeom M, Kim EY, Kim JH, Jung HS, Sohn Y. High Doses of Bupleurum falcatum Partially Prevents Estrogen Deficiency-Induced Bone Loss With Anti-osteoclastogenic Activity Due to Enhanced iNOS/NO Signaling. FRONT PHARMACOL. [Journal Article]. 2018 2018-01-20;9:1314.

76. Ozgocmen S, Kaya H, Fadillioglu E, Aydogan R, Yilmaz Z. Role of antioxidant systems, lipid peroxidation, and nitric oxide in postmenopausal osteoporosis. MOL CELL BIOCHEM. [Journal Article]. 2007 2007-01-01;295(1-2):45-52.

77. Banfi G, Iorio EL, Corsi MM. Oxidative stress, free radicals and bone remodeling. CLIN CHEM LAB MED. [Journal Article; Review]. 2008 2008-01-20;46(11):1550-5.

78. Chen K, Qiu P, Yuan Y, Zheng L, He J, Wang C, et al. Pseurotin A Inhibits Osteoclastogenesis and Prevents Ovariectomized-Induced Bone Loss by Suppressing Reactive Oxygen Species. THERANOSTICS. [Journal Article; Research Support, Non-U.S. Gov't]. 2019 2019-01-20;9(6):1634-50.

79. Agidigbi TS, Kim C. Reactive Oxygen Species in Osteoclast Differentiation and Possible Pharmaceutical Targets of ROS-Mediated Osteoclast Diseases. INT J MOL SCI. [Journal Article; Review]. 2019 2019-07-22;20(14).

80. Wang L, Ma R, Guo Y, Sun J, Liu H, Zhu R, et al. Antioxidant Effect of Fructus Ligustri Lucidi Aqueous Extract in Ovariectomized Rats Is Mediated through Nox4-ROS-NF-κB Pathway. FRONT PHARMACOL. [Journal Article]. 2017 2017-01-20;8:266.

81. Tu C, Wu DZ, Huang YS, Zhuang JS, Zeng JH, Xu P, et al. Oxidative Stress Contributes to Hyperalgesia in Osteoporotic Mice. J PAIN RES. [Journal Article]. 2020 2020-01-20;13:131-42.

82. Thi MM, Suadicani SO, Spray DC. Fluid flow-induced soluble vascular endothelial growth factor isoforms regulate actin adaptation in osteoblasts. J BIOL CHEM. [Journal Article; Research Support, N.I.H., Extramural]. 2010 2010-10-01;285(40):30931-41.

83. Schipani E, Maes C, Carmeliet G, Semenza GL. Regulation of osteogenesis-angiogenesis coupling by HIFs and VEGF. J BONE MINER RES. [Journal Article; Review]. 2009 2009-08-01;24(8):1347-53.

84. Thi MM, Iacobas DA, Iacobas S, Spray DC. Fluid shear stress upregulates vascular endothelial growth factor gene expression in osteoblasts. Ann N Y Acad Sci. [Journal Article]. 2007 2007-11-01;1117:73-81.

85. Senel K, Baykal T, Seferoglu B, Altas EU, Baygutalp F, Ugur M, et al. Circulating vascular endothelial growth factor concentrations in patients with postmenopausal osteoporosis. ARCH MED SCI. [Journal Article]. 2013 2013-08-30;9(4):709-12.

86. Helmrich U, Di Maggio N, Güven S, Groppa E, Melly L, Largo RD, et al. Osteogenic graft vascularization and bone resorption by VEGF-expressing human mesenchymal progenitors. BIOMATERIALS. [Journal Article; Research Support, Non-U.S. Gov't]. 2013 2013-07-01;34(21):5025-35.

87. Suresh S, de Castro LF, Dey S, Robey PG, Noguchi CT. Erythropoietin modulates bone marrow stromal cell differentiation. BONE RES. [Journal Article]. 2019 2019-01-20;7:21.

88. Li C, Shi C, Kim J, Chen Y, Ni S, Jiang L, et al. Erythropoietin promotes bone formation through EphrinB2/EphB4 signaling. J DENT RES. [Journal Article; Research Support, Non-U.S. Gov't]. 2015 2015-03-01;94(3):455-63.

89. Tsiftsoglou AS. Erythropoietin (EPO) as a Key Regulator of Erythropoiesis, Bone Remodeling and Endothelial Transdifferentiation of Multipotent Mesenchymal Stem Cells (MSCs): Implications in Regenerative Medicine. CELLS-BASEL. [Journal Article; Review]. 2021 2021-08-20;10(8).

90. Eggold JT, Rankin EB. Erythropoiesis, EPO, macrophages, and bone. BONE. [Journal Article; Research Support, N.I.H., Extramural; Research Support, U.S. Gov't, Non-P.H.S.; Review]. 2019 2019-02-01;119:36-41.

91. Xie H, Cui Z, Wang L, Xia Z, Hu Y, Xian L, et al. PDGF-BB secreted by preosteoclasts induces angiogenesis during coupling with osteogenesis. NAT MED. [Journal Article; Research Support, N.I.H., Extramural; Research Support, Non-U.S. Gov't]. 2014 2014-11-01;20(11):1270-8.

92. Horner A, Bord S, Kemp P, Grainger D, Compston JE. Distribution of platelet-derived growth factor (PDGF) A chain mRNA, protein, and PDGF-alpha receptor in rapidly forming human bone. BONE. [Journal Article; Research Support, Non-U.S. Gov't]. 1996 1996-10-01;19(4):353-62.

93. Zhang Z, Chen J, Jin D. Platelet-derived growth factor (PDGF)-BB stimulates osteoclastic bone resorption directly: the role of receptor beta. Biochem Biophys Res Commun. [Journal Article; Research Support, Non-U.S. Gov't]. 1998 1998-10-09;251(1):190-4.

94. Caplan AI, Correa D. PDGF in bone formation and regeneration: new insights into a novel mechanism involving MSCs. J ORTHOP RES. [Journal Article; Research Support, N.I.H., Extramural; Research Support, Non-U.S. Gov't; Research Support, U.S. Gov't, Non-P.H.S.]. 2011 2011-12-01;29(12):1795-803.

95. Peng Y, Wu S, Li Y, Crane JL. Type H blood vessels in bone modeling and remodeling. THERANOSTICS. [Journal Article; Research Support, Non-U.S. Gov't; Review]. 2020 2020-01-20;10(1):426-36.

96. Zheng X, Zhang Y, Guo S, Zhang W, Wang J, Lin Y. Dynamic expression of matrix metalloproteinases 2, 9 and 13 in ovariectomy-induced osteoporosis rats. EXP THER MED. [Journal Article]. 2018 2018-09-01;16(3):1807-13.

97. Inoue K, Mikuni-Takagaki Y, Oikawa K, Itoh T, Inada M, Noguchi T, et al. A crucial role for matrix metalloproteinase 2 in osteocytic canalicular formation and bone metabolism. J BIOL CHEM. [Journal Article; Research Support, N.I.H., Extramural; Research Support, Non-U.S. Gov't]. 2006 2006-11-03;281(44):33814-24.

98. Jiang L, Sheng K, Wang C, Xue D, Pan Z. The Effect of MMP-2 Inhibitor 1 on Osteogenesis and Angiogenesis During Bone Regeneration. Front Cell Dev Biol. [Journal Article]. 2020 2020-01-20;8:596783.

99. Andersen TL, Del COM, Kirkegaard T, Lenhard T, Foged NT, Delaissé JM. A scrutiny of matrix metalloproteinases in osteoclasts: evidence for heterogeneity and for the presence of MMPs synthesized by other cells. BONE. [Journal Article]. 2004 2004-11-01;35(5):1107-19.

100. Bernardo-Filho M, Sañudo B, Seixas A, Sá-Caputo D, Taiar R. Integrated Role of Nonpharmacological Interventions for Rehabilitation of Individuals with Musculoskeletal Disorders. BIOMED RES INT. [Editorial]. 2020 2020-01-20;2020:9493623.

101. Cackowski FC, Anderson JL, Patrene KD, Choksi RJ, Shapiro SD, Windle JJ, et al. Osteoclasts are important for bone angiogenesis. BLOOD. [Journal Article; Research Support, Non-U.S. Gov't]. 2010 2010-01-07;115(1):140-9.

102. Sivaraj KK, Adams RH. Blood vessel formation and function in bone. DEVELOPMENT. [Journal Article; Research Support, Non-U.S. Gov't; Review]. 2016 2016-08-01;143(15):2706-15.

103. Fei Y, Hurley MM. Role of fibroblast growth factor 2 and Wnt signaling in anabolic effects of parathyroid hormone on bone formation. J CELL PHYSIOL. [Journal Article; Research Support, N.I.H., Extramural; Review]. 2012 2012-11-01;227(11):3539-45.

104. Montero A, Okada Y, Tomita M, Ito M, Tsurukami H, Nakamura T, et al. Disruption of the fibroblast growth factor-2 gene results in decreased bone mass and bone formation. J CLIN INVEST. [Journal Article; Research Support, U.S. Gov't, P.H.S.]. 2000 2000-04-01;105(8):1085-93.

105. Kang F, Yi Q, Gu P, Dong Y, Zhang Z, Zhang L, et al. Controlled growth factor delivery system with osteogenic-angiogenic coupling effect for bone regeneration. J Orthop Translat. [Journal Article]. 2021 2021-11-01;31:110-25.

106. Clough E, Barrett T. The Gene Expression Omnibus Database. Methods Mol Biol. [Journal Article; Research Support, N.I.H., Intramural]. 2016 2016-01-20;1418:93-110.

107. Piñero J, Ramírez-Anguita JM, Saüch-Pitarch J, Ronzano F, Centeno E, Sanz F, et al. The DisGeNET knowledge platform for disease genomics: 2019 update. NUCLEIC ACIDS RES. [Journal Article; Research Support, Non-U.S. Gov't]. 2020 2020-01-08;48(D1):D845-55.

108. Wishart DS, Feunang YD, Guo AC, Lo EJ, Marcu A, Grant JR, et al. DrugBank 5.0: a major update to the DrugBank database for 2018. NUCLEIC ACIDS RES. [Journal Article; Research Support, Non-U.S. Gov't]. 2018 2018-01-04;46(D1):D1074-82.

109. Stelzer G, Rosen N, Plaschkes I, Zimmerman S, Twik M, Fishilevich S, et al. The GeneCards Suite: From Gene Data Mining to Disease Genome Sequence Analyses. Curr Protoc Bioinformatics. [Journal Article]. 2016 2016-06-20;54:1-30.

110. Rappaport N, Twik M, Plaschkes I, Nudel R, Iny ST, Levitt J, et al. MalaCards: an amalgamated human disease compendium with diverse clinical and genetic annotation and structured search. NUCLEIC ACIDS RES. [Journal Article; Research Support, Non-U.S. Gov't; Review]. 2017 2017-01-04;45(D1):D877-87.

111. Amberger JS, Hamosh A. Searching Online Mendelian Inheritance in Man (OMIM): A Knowledgebase of Human Genes and Genetic Phenotypes. Curr Protoc Bioinformatics. [Journal Article; Review]. 2017 2017-06-27;58:1-2.

112. Barbarino JM, Whirl-Carrillo M, Altman RB, Klein TE. PharmGKB: A worldwide resource for pharmacogenomic information. Wiley Interdiscip Rev Syst Biol Med. [Journal Article; Research Support, N.I.H., Extramural; Review]. 2018 2018-07-01;10(4):e1417.

113. Yu W, Clyne M, Khoury MJ, Gwinn M. Phenopedia and Genopedia: disease-centered and gene-centered views of the evolving knowledge of human genetic associations. BIOINFORMATICS. [Journal Article]. 2010 2010-01-01;26(1):145-6.

114. Chen X, Ji ZL, Chen YZ. TTD: Therapeutic Target Database. NUCLEIC ACIDS RES. [Journal Article]. 2002 2002-01-01;30(1):412-5.

115. Kanehisa M, Furumichi M, Tanabe M, Sato Y, Morishima K. KEGG: new perspectives on genomes, pathways, diseases and drugs. NUCLEIC ACIDS RES. [Journal Article; Research Support, Non-U.S. Gov't]. 2017 2017-01-04;45(D1):D353-61.

116. Du J, Yuan Z, Ma Z, Song J, Xie X, Chen Y. KEGG-PATH: Kyoto encyclopedia of genes and genomes-based pathway analysis using a path analysis model. MOL BIOSYST. [Journal Article; Research Support, Non-U.S. Gov't]. 2014 2014-07-29;10(9):2441-7.

117. Consortium TGO. The Gene Ontology Resource: 20 years and still GOing strong. NUCLEIC ACIDS RES. [Historical Article; Journal Article; Research Support, N.I.H., Extramural; Research Support, Non-U.S. Gov't]. 2019 2019-01-08;47(D1):D330-8.

118. Szklarczyk D, Gable AL, Lyon D, Junge A, Wyder S, Huerta-Cepas J, et al. STRING v11: protein-protein association networks with increased coverage, supporting functional discovery in genome-wide experimental datasets. NUCLEIC ACIDS RES. [Journal Article; Research Support, N.I.H., Extramural; Research Support, Non-U.S. Gov't; Research Support, U.S. Gov't, Non-P.H.S.]. 2019 2019-01-08;47(D1):D607-13.
